# Replication differences of SARS-CoV-2 lineages may arise from unique RNA replication characteristics and nucleocapsid protein expression

**DOI:** 10.3389/fcimb.2025.1582137

**Published:** 2025-07-11

**Authors:** Isadora Alonso Corrêa, Marcos Romário Matos de Souza, Gustavo Peixoto Duarte da Silva, Anna Beatriz Sampaio Vianna Macedo Pimentel, Pedro Telles Calil, Marcela Sabino Cunha, Diana Mariani, Rodrigo de Moraes Brindeiro, Sara Mesquita Costa, Maria Clara da Costa Simas, Victor Akira Ota, Elisa Cavalcante Pereira, Marilda Mendonça Siqueira, Paola Cristina Resende, Rafael Mello Galliez, Debora Souza Faffe, Rosane Silva, Terezinha Marta Pereira Pinto Castiñeiras, Amilcar Tanuri, Luciana Jesus da Costa

**Affiliations:** ^1^ Laboratório de Genética e Imunologia das Infecções Virais, Departamento de Virologia, Instituto de Microbiologia Paulo de Góes, Universidade Federal do Rio de Janeiro (UFRJ), Rio de Janeiro, Brazil; ^2^ Laboratório de Virologia Molecular, Departamento de Genética, Instituto de Biologia, Universidade Federal do Rio de Janeiro (UFRJ), Rio de Janeiro, Brazil; ^3^ Laboratório de Metabolismo Macromolecular Firmino Torres de Castro, Instituto de Biofísica Carlos Chagas Filho, Universidade Federal do Rio de Janeiro (UFRJ), Rio de Janeiro, Brazil; ^4^ Núcleo de Enfrentamento e Estudos de Doenças Infecciosas Emergentes e Reemergentes (NEEDIER), Universidade Federal do Rio de Janeiro (UFRJ), Rio de Janeiro, Brazil; ^5^ Laboratório de Vírus Respiratórios, Exantemáticos, Enterovírus e Emergência Viral, Instituto Oswaldo Cruz, Fundação Oswaldo Cruz (Fiocruz), Rio de Janeiro, Brazil; ^6^ Departamento de Doenças Infecciosas, Faculdade de Medicina, Universidade Federal do Rio de Janeiro (UFRJ), Rio de Janeiro, Brazil

**Keywords:** SARS-CoV-2, variants, replication, virus-cell interaction, RNA replication, nucleocapsid protein

## Abstract

**Introduction:**

The COVID-19 pandemic was characterized by the sequential introduction and circulation of distinct SARS-CoV-2 variants, which presented differences in transmission capacity and pathogenicity. However, the relationship between these differences and the replicative capacity of these variants remains to be determined. Our research aimed to compare the biological traits of the SARS-CoV-2 lineages B.1.1.33, and variants Zeta (P.2), Gamma (P.1/P.1.*), Delta (B.1.617.2/AY.*), and Omicron (BA.1).

**Methods:**

We employed three different cellular models susceptible to viral infection to demonstrated the differences in virus binding, entry and total RNA production through RT-qPCR assay and viral infectious progeny by plaque assay. The RNA replication was evaluated by dsRNA immunofluorescence and the viral protein production by western blotting analysis. NGS and RT-qPCR analysis were also used in competition experiments to verify the viral variants dynamic in cell culture.

**Results:**

We found that the differences in viral replication varied according to the cell type, with Omicron BA.1 exhibiting the lowest replication capacity in human pulmonary cells. Additionally, we demonstrated the occurrence of nucleocapsid proteoforms generated during infection and differences in size and number of sites of viral RNA replication for each virus.

**Conclusion:**

These data suggest that factors beyond the initial stages of virus entry influence the efficiency of viral replication among different SARS-CoV-2 variants. Thus, our study underscores the significance of RNA replication and the role of nucleocapsid proteins in shaping the replicative characteristics of SARS-CoV-2 variants.

## 1 Introduction

The Severe Acute Respiratory Syndrome Coronavirus 2 (SARS-CoV-2) was identified in December 2019 and is responsible for the coronavirus disease 2019 (COVID-19) pandemic. Despite its lower mutation rates compared to other RNA viruses, some factors contribute to the accumulation/selection of mutations within the viral genome such as: the widespread virus circulation, independent introduction events, host adaptive immune pressure, persistent infections, and the high immunization level (Plante et al., 2021; van Dorp et al., 2020; Gupta et al., 2024). The first major mutation that altered viral dynamics was the substitution of the Spike (S) glycoprotein from D to G, which occurred in March 2020 and rapidly overcame the original Wuhan virus (Korber et al., 2020). Studies demonstrated that G614 viruses replicate more than D614 in the superior airways and, in hamster models and competition assays, could overcome D614 viruses even when these were the major population (Hou et al., 2020; Plante et al., 2020). The diversity of SARS-CoV-2 genomes led to the WHO´s classification as variants of interest (VOIs) and variants of concern (VOCs), which possess genetic changes that could affect viral transmissibility, disease severity, and immune escape (World Health Organization, 2023).

The VOCs Alpha (B.1.1.7) and Beta (B.1.351) emerged in the United Kingdom and South Africa, respectively, in 2020. The VOC Delta (B.1.617.2/AY.*), first described in October 2020 in India, quickly spread around the world and accumulated more mutations in the S gene than the previous variants (Campbell et al., 2021). However, in November 2021, Delta was replaced by a new VOC that emerged in Botswana and South Africa named Omicron (BA.1). In January 2022, Omicron BA.1 dominated the pandemic scenario even in countries with high vaccination rates (Hodcroft, 2021; Viana et al., 2022). This variant presented over 30 substitutions in the S protein (Dhama et al., 2023) and currently continues to evolve (Hodcroft, 2021). By the time of its emergency, these variants were classified as VOCs; however, until April 2025, the WHO classification did not consider any SARS-CoV-2 variants as VOCs and only one variant (JN.1) as VOI (World Health Organization, 2025).

After the first case was confirmed in Brazil in late February 2020, multiple viral introductions were documented in the country, with the predominance of two major lineages, B.1.1.28 and B.1.1.33 (Candido et al., 2020). From this lineage derived the variants Gamma P.1 and P.1.* in December 2020 and, by January 2021, became the most prevalent variant in the country and was associated with a high mortality rate (Naveca et al., 2021a), high viral loads, and transmissibility (Faria et al., 2021; Naveca et al., 2021b). Zeta (P.2) variant, also derived from B.1.1.28 and B.1.1.33, emerged around October 2020 and was associated with an increase in infection rates in the country (Voloch et al., 2021; Genomahcov, 2021).

SARS-CoV-2 circulation is highly dynamic, and the mechanisms driving the spread and infectiousness of viral variants remain unclear. For Delta and Gamma, lower cycle thresholds (Ct) values were reported in patients positive for SARS-CoV-2 (Li et al., 2022; Moreira et al., 2021), and Delta was associated with increased disease severity (Ong et al., 2021). Despite its high transmissibility, several studies indicate that Omicron BA.1 has impaired replication in pulmonary cells (Shuai et al., 2022; Hui et al., 2022a; Hénaut et al., 2023), resulting in lower pathogenicity in mouse and hamster models than previous variants (Halfmann et al., 2022; Peacock et al., 2022; Banete et al., 2025). Also, nasal samples from Omicron-infected patients showed lower viral loads when compared to Delta infections (Laitman et al., 2022; Sentis et al., 2022). However, Omicron presents faster replication rates in the nasal epithelium and human bronchi (Hui et al., 2022a; Peacock et al., 2022).

A common feature of these variants is the escape from neutralizing sera from previously infected or post-vaccinated individuals with a reduction in neutralization capacity for Alpha, Delta, Gamma, and Omicron reported in individuals that received one or two doses of different vaccines (de Souza et al., 2021a; Lopez Bernal et al., 2021; Evans et al., 2021; Zeng et al., 2021) as for individuals previously infected by SARS-CoV-2 (Carreño et al., 2021; Diamond et al., 2021; de Souza et al., 2021a). Thus, the driving force behind the emergence of new variants and the replacement of previously circulating ones is the elicited immune response against SARS-CoV-2. However, the replication characteristics of the continuously emerging SARS-CoV-2 variants will also influence virus transmissibility and spread rates, and consequently, the impact of the variant on the human population. Differences in replication characteristics are expected for different variants based on the presence of various mutations in critical genes. Thus, studying the biological characteristics and replication dynamics of the viruses that have circulated since the beginning of the pandemic will identify crucial traits for viral adaptation, enhance our understanding of this pandemic virus and its ability to adapt to new scenarios, and inform more effective strategies to prevent the emergence of new variants. The present study compares the biological characteristics of SARS-CoV-2 variants B.1.1.33, Zeta, Gamma, Delta, and Omicron BA.1. Our work indicates that Omicron BA.1 has the lowest replication compared to previous variants in a human pulmonary cell line. Besides, our results highlight diverse aspects of viral replication for each variant, including binding, entry, RNA replication, and viral protein production. Collectively, we demonstrate the importance of the RNA replication step and the role of Nucleoprotein (N) in shaping the replicative characteristics of the SARS-CoV-2 variants.

## 2 Materials and methods

### 2.1 Viral isolation and viral stocks

SARS-CoV-2 lineages B.1.1.33, Zeta, Gamma, and Delta were isolated from nasopharyngeal swab samples collected from individuals attending the Núcleo de Enfrentamento e Estudo de Doenças Infecciosas Emergentes e Reemergentes. Samples were collected at the following periods: B.1.1.33, August 2020; Zeta, November 2020; Gamma, March 2021; and Delta, August 2021.

From these SARS-CoV-2 positive samples, the viral transport medium (VTM) was used to infect Vero E6 cells. Briefly, cells were seeded in 6-well plates overnight in DMEM with 10% FBS to achieve 70% confluence. Then, 250 µL of VTM diluted in 250 µL DMEM without FBS was added for one hour and after incubation, the inoculum was replaced by DMEM with 10% FBS, and cells were maintained at 37°C and 5% CO2. The culture was observed until visualization of the cytopathic effect (CPE). After this first passage, passages were performed in Vero/hACE-2/hTMPRSS2, at the same conditions described above. Viral stocks were generated from the second passage, representing viruses from the third passage. After viral isolation, viral stocks were generated by infection of 2.0 x 10^6^ Vero/hACE-2/hTMPRSS2 (2.0 x 10^6^) with an MOI of 0.01 of each SARS-CoV-2 variant. Infected cells were cultured for 72 h when, the supernatant from each infection was harvested and filtered through a 0.22 µM filter to remove cellular debris. The stocks were aliquoted and stored at -80°C. The Omicron (BA.1) variant was isolated in December 2021, and kindly given by professors Edison Luiz Durigon (USP), Ester Sabino (IMT-SP), Fernando Spilki (FEEVALE-SC), and João Renato Rebello Pinho (HIAE). The Omicron BA.1 following passages and stock were conducted as described above.

### 2.2 Biosafety

The study was approved by the ethical committee (CAAE-30161620.0.1001.5257) and all the experiments using infectious particles were performed in a BSL-3 laboratory.

### 2.3 Sequencing of viral stocks

All viral stocks were sequenced after cell passage to confirm viral lineage and the emergence of non-defining lineage mutations. The GISAID (GISAID, 2024) accession numbers are summarized in **Table 1**. The complete coverage of the SARS-CoV-2 genome was obtained through NGS, using the Ion AmpliSeq SARS-CoV-2 Research Panel (ThermoFisher Scientific, Massachusetts, USA). The multiplex amplification reaction was conducted using 10 µl of the cDNA according to the manufacturer’s instructions for 21 cycles of the multiplex RT-PCR-specific SARS-CoV-2 primers from the panel. Library quantification was performed using the Ion Library TaqMan™ Quantitation Kit (ThermoFisher Scientific, Massachusetts, USA – cat # 4468802). Libraries were pooled at a concentration of 50 pM, and the emulsion PCR and enrichment reactions were conducted on the Ion Chef™ system (ThermoFisher Scientific, Massachusetts, USA) using Ion 510, 520 & 530 kits (ThermoFisher Scientific, Massachusetts, USA – cat # A34461). Using a 530 chip, the sequencing reaction was performed on the Ion S5™ System genetic sequencer (ThermoFisher Scientific, Massachusetts, USA). Reads generated were mapped to the SARS-CoV-2 reference genome Wuhan (NCBI GenBank accession number MN908947) using the Ion Browser software included in the Torrent Suite 5.18.1. Virus genome assembly was carried out with the IRMA plugin v.1.3.0.2. Lineage was accessed using the web tool Nextclade version 2.14.1 (Nextstrain project) (Aksamentov et al., 2021) through fasta files generated from NGS sequencing.

**Table 1:** Accession number of the SARS-CoV2 sequenced viral stocks at the EpiCoV database from GISAID.

SARS-CoV-2 variant and lineage	Type cell and passage Number	GISAID number
B.1.1.33	original sample (VTM)	EPI_ISL_11836071
B.1.1.33	Vero/hACE-2/hTMPRSS2- 4	EPI_ISL_18433588
Zeta (P.2)	Vero/hACE-2/hTMPRSS2- 4	EPI_ISL_18433589
Gamma (P.1)	Vero E6 - 2	EPI_ISL_18433882
Gamma (P.1)	Vero/hACE-2/hTMPRSS2- 3	EPI_ISL_18433590
Gamma (P.1)	Vero/hACE-2/hTMPRSS2- 4	EPI_ISL_18433591
Delta	Vero/hACE-2/hTMPRSS2- 3	EPI_ISL_18433883
Delta	Vero/hACE-2/hTMPRSS2- 4	EPI_ISL_18433592
Omicron (BA.1)	original sample (VTM)	EPI_ISL_7699344
Omicron (BA.1)	Vero/hACE-2/hTMPRSS2- 1	EPI_ISL_18433884
Omicron (BA.1)	Vero/hACE-2/hTMPRSS2- 2	EPI_ISL_18433885
Omicron (BA.1)	Vero/hACE-2/hTMPRSS2- 3	EPI_ISL_18433886
Omicron (BA.1)	Vero/hACE-2/hTMPRSS2- 4	EPI_ISL_18433887

### 2.4 Cell cultures

African green monkey kidney cells - VeroE6, (ATCC CRL-1586) and VeroE6 cells expressing human transmembrane protease serine 2 and human angiotensin-converting enzyme 2-Vero/hACE-2/hTMPRSS2 cells (NR-54970) were maintained in Dulbecco’s Modified Eagle Medium (DMEM), with 4.5 g/L D-glucose (Gibco – cat #11995-073) supplemented with 10% fetal bovine serum (FBS) (Gibco – cat #12657-029), 100 U/mL penicillin, and 100 μg/mL of streptomycin (Gibco – cat #15140-122). Human lung adenocarcinoma epithelial cells - Calu-3, (ATCC HTB-55) were cultured DMEM with 1.0 g/L D-glucose (Gibco – cat #12320-032) supplemented with 10% FBS. All cells were maintained at 37°C and 5% CO2 and routinely tested for mycoplasma.

### 2.5 Viral binding and entry assay

For viral binding assays VeroE6, and Vero/hACE-2/hTMPRSS2 (2,0 x 10^6^ cells), and Calu-3 cells (3,0 x 10^6^ cells) were plated in 24-well plates and inoculated with a fixed quantity of SARS-CoV-2 genomic viral RNA copies, diluted in non-supplemented DMEM, at 4°C for 1h. After the viral adsorption period, the monolayers were washed with cold 1X PBS to remove unattached particles, followed by harvesting cells in the TRIzol reagent (Invitrogen – cat #15596026). For virus entry, the same amount of VeroE6, and Vero/hACE-2/hTMPRSS2 and Calu-3 cells were also infected at the same way as described for the binding assay. After the viral adsorption period, the monolayers were washed with cold 1X PBS to remove unattached particles, followed by the addition of 10% SBF-supplemented DMEM and incubation at 37°C with 5% CO2 for another 1 h. After this 1 h entry period, cells were collected in TRIzol. Both binding and entry experiments were performed twice for each cell line, with three replicates for each SARS-CoV-2 variant in each experiment.

Total RNA from both experiments was extracted following the TRIzol manufacturer’s instructions. Detection and quantification of SARS-CoV-2 was made by RT-qPCR using the Detection Kit for 2019 Novel Coronavirus (2019-nCoV) RNA (DaAnGene - cat #DA-930). Virus binding was calculated as the number of viral genomic RNA copies, derived from Ct values using a standard curve obtained with SARS-CoV-2 synthetic RNA, obtained after incubation at 4°C. Virus entry was calculated as the ΔCt of 37°C and 4°C incubation periods.

### 2.6 Viral kinetics assay

Viral replication kinetics were performed in 24-well plates seeded with 4,0x10^6^ cells for VeroE6 and Vero/hACE-2/hTMPRSS2, and with 3,5x10^6^ for Calu-3 cells. Monolayers were infected with SARS-CoV-2 variants (MOI of 0.1). Supernatants and cells were collected after 12, 24, 48, and 72 hours post infection (hpi) for Vero cells and up to 96 hpi for Calu-3 cells. The samples were stored at -80°C. Supernatants were used for viral titration by plaque assay, and RNA extraction with Bio Gene (Bioclin – cat #K204-4). Cells were treated with TRIzol (Invitrogen – cat #15596026)) for RNA extraction.

### 2.7 Viral titration

Stocks and experiments supernatants were titrated as follows. Vero/hACE-2/hTMPRSS2 cells were plated overnight in 12-well plates to achieve 90% confluence. Cell media was then replaced by 200 µL of 10-fold serial dilutions of each sample for infection. After one hour of adsorption, media was replaced by DMEM (Gibco – cat # 12100-046) with 1% FBS, 100 µg/mL penicillin, 100 µg/mL streptomycin, and 1.4% carboxymethylcellulose (Sigma Aldrich – cat #C5678), followed by incubation for three days at 37°C with 5% CO2. After 3 days of incubation, cells were fixed with 5% formaldehyde and stained with 1% crystal violet in 20% methanol for plaque visualization and quantification. Viral titers were expressed as plaque-forming units (PFU) per milliliter.

### 2.8 cDNA synthesis, genomic and subgenomic detection

For genomic SARS-CoV-2 RNA detection, real-time RT-qPCR reactions were performed using the Detection Kit for 2019 Novel Coronavirus (2019-nCoV) RNA (DaAnGene - cat #DA-930) for detection of N and ORF genes according to manufacturer’s instructions. For sub-genomic RNA (sgRNA) and *gapdh* detection, cDNA synthesis was performed with the High-Capacity cDNA Reverse Transcription kit (Applied Biosystems – cat # 4368814) priming with random hexamers. N sgRNA was detected by TaqMan™ Universal PCR Master Mix (Applied Biosystems – cat # 4304437) using the forward primer 5’ CGA TCT CTT GTA GAT CTG TTC TCT AAA CGA ACT TAT GTA CTC 3’; reverse primer 5’ ATA TTG CAG CAG TAC GCA CAC A 3’; and probe FAM-5’ ACA CTA GCC ATC CTT ACT GCG CTT CG 3’-IowaBlack. Reactions were cycled as follows: 95°C for 2 minutes; 45 cycles of 95°C for 15 seconds, and 53°C for 30 seconds. Sample analysis was performed using a threshold of 0.01.

The *gapdh* was detected by Power SYBR Green PCR master mix reagent (Applied Biosystems – cat # 4309155) using the forward primer 5’ GTG GAC CTG ACC TGC CGT CT 3’ and reverse primer 5’ GGA GGA GTG GGT GTC GTC GT 3’. Reactions were cycled at: 95°C for 2 minutes; 40 cycles of 95°C for 15 seconds; and 60°C for 1 minute following the melting cycle. Intracellular sgRNA RNA levels were normalized by the *gapdh* cycle threshold values. All reactions were performed at AriaMx Real-time PCR System (Agilent Technologies).

### 2.9 SARS-CoV-2 RNA copy number quantification

To determine viral RNA copy number, a dose-response curve was constructed by simple linear regression. We used a positive control with known gene copy number (2019- nCoV_N_Positive Control, IDT - cat. #10006625) made of an *in vitro* transcribed and purified plasmid DNA target that contains 200,000 copies of gene N per microliters.

### 2.10 Cell viability assay

Supernatants from non-infected and infected cell cultures were harvested from all the incubation time points and used for determination of the percentage of cell survival using LDH-Glo™ Cytotoxicity Assay (Promega – cat #J2381) kit following the manufacturer’s instructions.

### 2.11 Competition assay

Vero/hACE-2/hTMPRSS2 (4,0 x 10^6^ cells/plate) and Calu-3 cells (3,5 x 10^6^ cells) were seeded in 48-well plates and co-infected with three different proportions of SARS-CoV-2 variants: 1:1, 1:9 and 9:1 resulting in a final MOI of 0.1. The combination of variants used in this assay was as follows: Gamma and Omicron, Delta and Omicron, Zeta and Gamma, and Gamma and Delta. The culture supernatants were harvested after 24 and 48 h of infection and stored at -80°C for further RNA extraction. Total RNA was extracted from supernatants with Bio Gene (Bioclin - cat #K204-4) and then used for major and minor viral population detection by NGS or RT-qPCR. For RT-qPCR detection, primers and probes were designed to detect two variant defining mutations: a deletion at NSP6 gene that occurs exclusively at the Gamma variant; and the deletion of amino acids 69 and 70 at the S gene that occurs in the Alpha and Omicron BA.1 variants. In addition, primers and probes for envelope (E) and nucleocapsid (N) targets were used for total RNA quantification. Samples double negative for NSP6 and 69–70 deletions were classified as Delta or Zeta, while samples positive only for NSP6 deletion were classified as Gamma. The percentage of each variant was calculated as the ΔCt of the specific target minus the nucleocapsid target.

### 2.12 SARS-CoV-2 whole genome sequencing of the competition assay

For the competition assays revealed by NGS, the SARS-CoV-2 whole genome was recovered using Illumina COVIDSeq Test (Illumina, CA, USA) and the ARTIC 4.1v primer set (https://artic.network/). The final library was submitted to the MiSeq platform (Illumina, CA, USA) to achieve 2000x coverage and the results obtained in the Illumina Basespace Sequence Hub. The reads were assembled using the reference genome MN985325.1 (USA/WA1/2020). Bioinformatic analysis was performed with Illumina^®^ DRAGEN COVID Lineage App (version 3.5.12) and assembled by ViralFlow 1.0.060 (Dezordi et al., 2022). The ViralFlow pipeline can generate a report that includes the analysis of minor variants. For lineage assignment, the Pango Lineage tool was used. Consensus fasta sequences were uploaded to the EpiCoV database in GISAID.

### 2.13 Immunofluorescence

Calu-3 cells were cultured overnight onto coverslips followed by infection with SARS-CoV-2 variants (at an MOI of 0.1). Then, culture supernatants were harvested at 8- and 24-hours post-infection and cells were fixed with 4% PFA in PBS for 15 min and permeabilized with 0.1% Triton X-100 (Sigma-Aldrich – X100-5ML) with 3% BSA (Sigma-Aldrich – A1906) in PBS for 20 min. Cells were incubated with J2 anti-dsRNA IgG2a monoclonal antibody (Scicons – cat #RNT-SCI-10010200) diluted 1:1000 in 3% BSA in PBS for 1 h at 4°C. Then, cells were washed and incubated with AlexaFluor 488-conjugated anti-mouse IgG (Thermo Scientific – cat #A28175.) diluted 1:1000 in PBS for 40 min. Cells were washed and incubated with DAPI staining (Invitrogen – cat #D1306) for 10 min. Coverslips were mounted with ProLong™ Gold Antifade Reagent (Thermo Scientific – cat #P36934) and imaged on a Zeiss 817 LSM 710 confocal microscope. Images were obtained with the ELYRA Zeiss PS.1 confocal microscope. To obtain the percentage of dsRNA positive cells in each experimental condition, fluorescence positive cells were manually counted using the cell count tool (multi-point) on the Image J software (version 2.14.q.54f). To obtain the Average J2 puncta 30 random cells from 25 different fields were manually selected to generate a unique picture using the tile scan tool at ELYRA Zeiss PS.1 confocal microscope. Automate analyses, using the IMAGE J protocol, were applied to quantify total J2 puncta. Average quantification data of dsRNA puncta area in μm2 was obtained by Image J automatic area measurement of 181–684 cells in 25 different fields.

### 2.14 Western blot

Whole infected-cell lysates were collected with RIPA buffer (10 mM Tris-Cl [pH 8.0]; 1 mM EDTA; 0.5 mM EGTA; 1% Triton X-100; 0.1% sodium deoxycholate; 0.1% SDS; 140 mM NaCl) added with 0.1% protease inhibitors cocktail (Sigma Aldrich – cat #P8340). Samples were loaded in 4%-20% Mini-Protean gels (Bio-Rad, cat #4561093). The following antibodies were used for viral protein analysis: SARS-CoV-2 anti-Spike and anti-N protein (Cell Signaling - cat #569996 and #33336, respectively); and anti-β-actin (Sigma – cat #a2228); anti-rabbit HRP (Cell Signaling – cat #70745); and anti-mouse HRP (Thermo Scientific – cat #31436).

### 2.15 Quantification and statistical analysis

Statistical assessments were conducted to compare two experimental groups via unpaired multiple t-tests. For comparisons involving three or more experimental groups, One-way ANOVA followed by Dunnett’s multiple comparisons or Two-way ANOVA followed by Bonferroni’s multiple comparisons test was employed. Differences were considered statistically significant when p<0.05. Data analysis was executed utilizing GraphPad Prism v 9.4.1 for Windows, GraphPad Software, www.graphpad.com .

## 3 Results

### 3.1 Distinct waves of SARS-CoV-2 infection in Brazil

**Figure 1:**
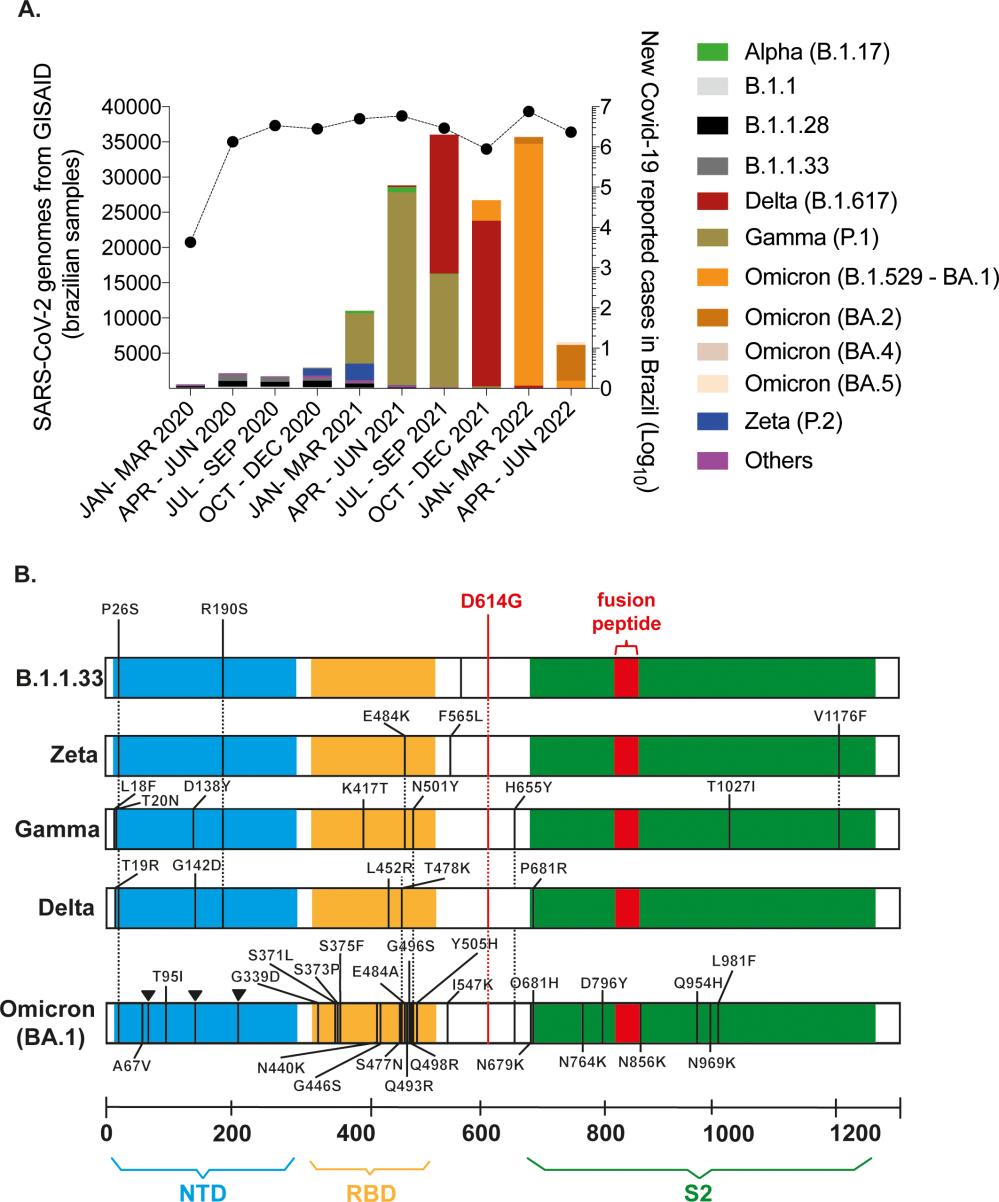
SARS-CoV-2 variants were associated with distinct waves of infections in Brazil. **(A)** Sequences derived from NGS sequencing of SARS-CoV-2 Brazilian samples were compiled by Fiocruz Genomic Network from January 2020 to June 2022. The left y-axis indicates the absolute number of deposited sequences. Each color represents a different viral lineage. The right y-axis shows the total number of Covid-19 cases in Brazil registered by the Covid global data from WHO during the same period (black line). **(B)** Spike (S) region representation for B.1.1.33, Zeta, Gamma, Delta, and Omicron (BA.1) genomes obtained by NGS sequencing of viral stocks generated after SARS-CoV- 2 isolation. The defining mutations of each lineage are indicated according to protein domains: N-terminal domain (NTD) in blue; receptor binding domain (RBD) in yellow; S2 subunit in green; and the fusion peptide in red.

Distinct waves of infection occurred in Brazil since February 2020, marking the emergence, circulation, and extinction of SARS-CoV-2 variants. Analysis of sequencing data obtained from Brazilian samples and compiled by Fiocruz Genomic Surveillance Network (Genomahcov, 2021) demonstrated that lineages B.1.1.28 and B.1.1.33 were present at the pandemic`s beginning and co-circulated predominantly up to September 2020. At that time, the Zeta variant emerged and prevailed being replaced by Gamma. Gamma rapidly dominated the Brazilian scenario, causing a second wave of infections that began in March 2021 (**Figure 1A**). After Gamma’s ascendence, the Delta variant was introduced in July 2021. Delta remained prevalent from October to December 2021 despite a decrease in confirmed cases. However, the introduction of Omicron (BA.1) caused a fourth wave of infections, leading to the highest number of reported cases until June 2022 (**Figure 1A**).

Our group continuously isolated and characterized the main circulating SARS-CoV-2 lineages from samples of patients attending NEEDIER. B.1.1.33, Zeta, Gamma, and Delta viruses were successfully isolated in VeroE6 cells. NGS sequencing was performed for these variants along with BA.1, and characteristic polymorphisms at the S gene and other genomic regions confirmed viral lineages (**Figure 1B**; **Supplementary Figure 1**). Isolates were serially passed up to passage 4. Viral stocks used in the experiments described here were generated from passage 2. Few mutations were selected during cell culture passages in variants Gamma (Orf1a and ORF3a), Delta (Orf1a), and BA.1 (Orf1a and Spike RBD domain); no mutations were selected within the furin-cleavage site in S glycoprotein (_681_PRRA_684_) during passages. Delta and BA.1 presented the signature substitutions P681R and P681H, respectively (**Supplementary Figure 1**). Although the deletion of the _675_QTQN_679_ motif upstream of the furin-cleavage has been commonly observed in *in vitro*-obtained viral stocks (Vu et al., 2022), no mutations or deletions were observed during the viral passages of our viral stocks (**Supplementary Figure 1**).

### 3.2 The advantages in the binding step did not correlate with the entry capacity of SARS-CoV-2 variants

**Figure 2:**
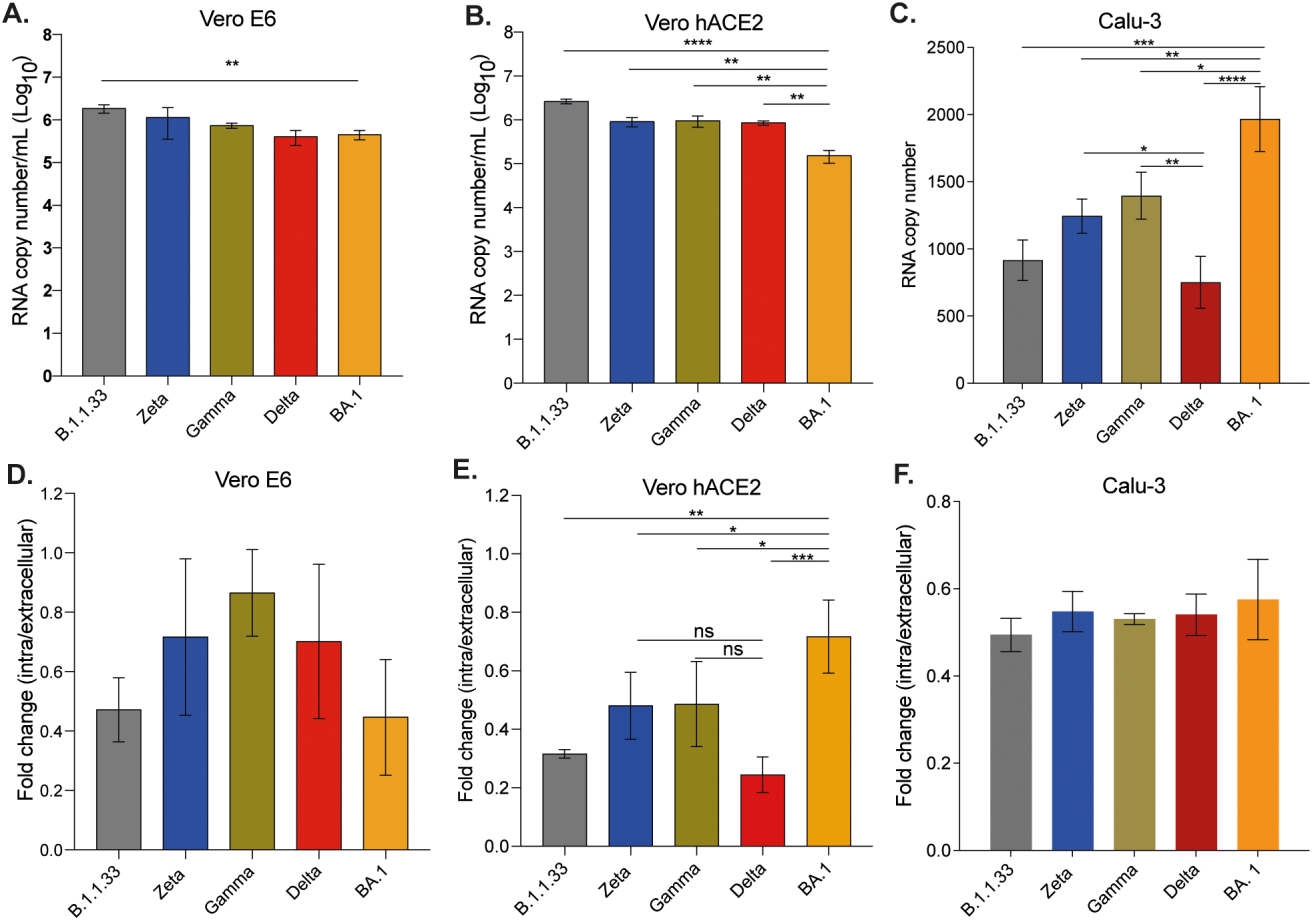
SARS-CoV-2 variants presented differential binding and entry capacities. Vero E6 **(A)**, Vero/hACE-2/hTMPRSS2 **(B)**, and Calu-3 **(C)** cells were incubated with the same copy number of RNA of each viral variant for 1 hour at 4°C to allow viral binding. After adsorption, cells were harvested for RNA extraction and SARS-CoV-2 detection and quantification by RT-qPCR. For entry assay, Vero E6 **(D)**, Vero/hACE- 2/hTMPRSS2 **(E)**, and Calu-3 **(F)** cells were incubated for 1 hour at 4°C and after adsorption, the inoculum was replaced by DMEM with 10% FBS and cells were incubated at 37°C with 5% CO2 for one more hour to allow entry. The efficiency of viral entry was calculated by the ΔCt between cellular lysates obtained after incubation at 37°C and 4°C. Data are shown as mean ± s.d. n=3. Statistical analysis was performed using one-way analysis of variance (ANOVA) followed by Dunnett´s multiple comparisons test using GraphPad Prism v9.4.1 for Windows (GraphPad Software, www.graphpad.com). p<0.05 was considered statistically significant. *p<0.05; **p<0.01; ***p<0;001; ****p<0.0001. Graphs not showing asterisks had non-significant p-values. All p-values are available in **Supplementary Table 1**.

Although all variants efficiently bound to the surface of VeroE6 and Vero/hACE-2/hTMPRSS2 cells, B.1.1.33 presented the highest binding capacity when compared to Gamma, Delta, and BA.1 (**Figures 2A, B** - top) in Vero/hACE-2/hTMPRSS2 cells. When compared to all other variants, BA.1 had the lowest ability to bind to Vero/hACE- 2/hTMPRSS2 cells (**Figure 2B**). However, BA.1 bound more efficiently to Calu-3 cells than all other variants, followed by Gamma (**Figure 2C**).

However, the greater binding ability observed for some variants in certain cell lines did not correlate with their entry capacity. For Vero E6 cells, despite the greater binding efficiency of B.1.1.33, there was no difference in entry among all viral variants analyzed (**Figure 2D**). On the other hand, despite the lower binding capacity of BA.1 on Vero/hACE-2/hTMPRSS2 cells, this variant presented the highest entry capacity compared to all other variants analyzed (**Figure 2E**). In this cell line, the Delta variant presented significantly lower entry capacity compared to Zeta, Gamma, and BA.1 (**Figure 2E**). In Calu-3 cells, however, no difference in entry ability was observed amongst the variants (**Figure 2F**). The expression of ACE-2 on Vero/hACE-2/hTMPRSS2 cells was similar to Calu-3 (**Supplementary Figure 2**). According to our results, the SARS-CoV-2 efficiency of binding and entry did not depend on levels of ACE-2 expression or the presence of TMPRSS2. In general, the native SARS-CoV-2 variants bound with the same efficiency to the surface of these cell lines, and a difference in entry was only observed in the cell line that heterologously expressed TMPRSS2 and ACE-2.

### 3.3 SARS-CoV-2 BA.1 had the highest replication capacity both in Vero-hACE and Vero E6 cells

**Figure 3:**
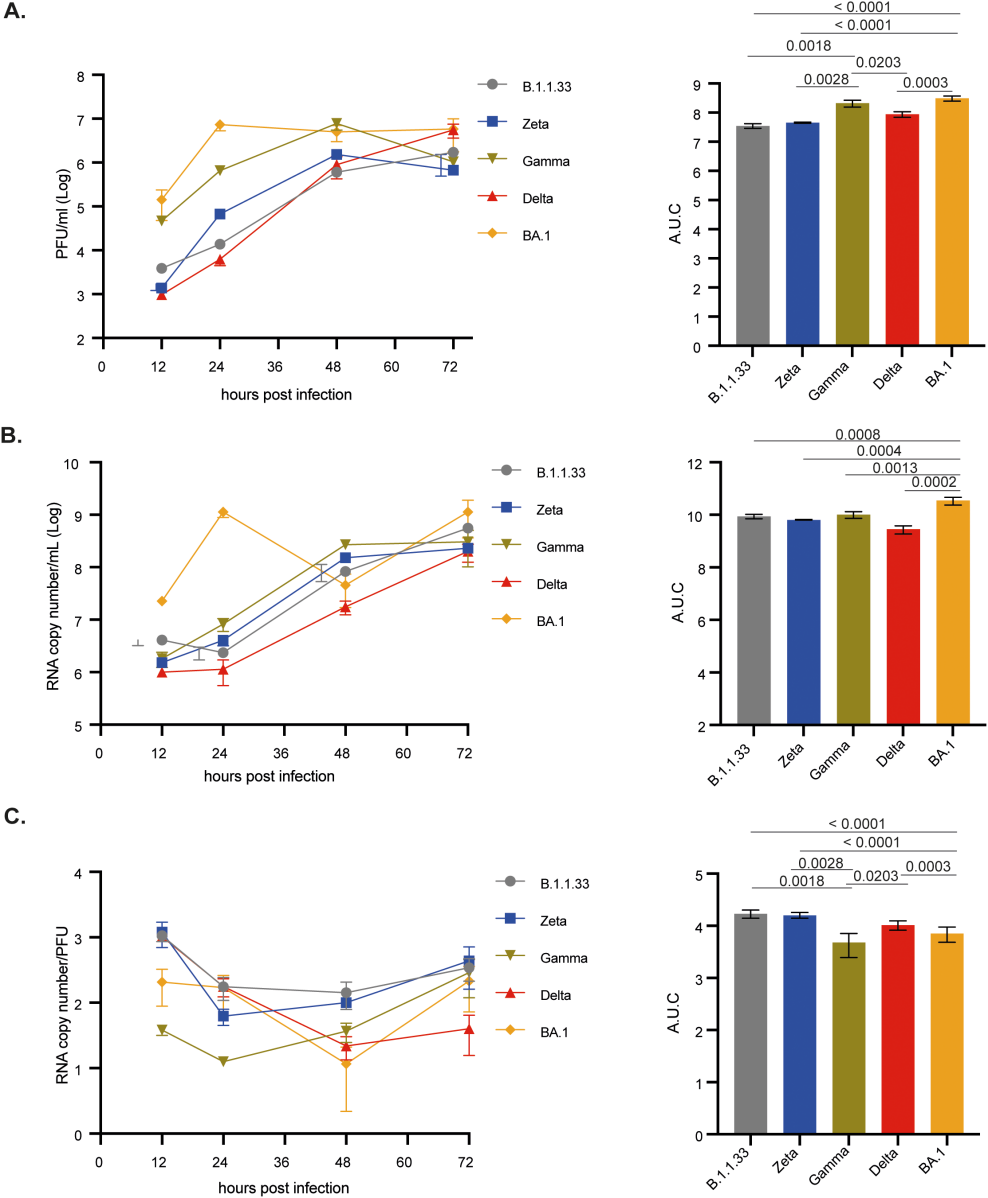
BA.1 variant showed greater infection capacity in Vero-hAce2/h-TMPRSS2 cells. Cells were infected with viral variants at MOI 0.1 and incubated at 37°C with 5% CO2 for 12, 24, 48, or 72 hours to evaluate viral replication kinetics. **(A)** Infectious viral progeny quantification by plaque assay at indicated time points. **(B)** Viral RNA copy number quantification by RT-qPCR at indicated time points. **(C)** Particle to plaque-forming unit ratio (P:PFU) obtained as a measurement of viral infectivity. Viral titers **(A)**, RNA copy numbers **(B)**, and P:PFU **(C)** are also depicted as areas under the curve (AUC). Data are shown as mean ± s.d. n=3. Statistical analysis of the AUC was performed using one-way ANOVA followed by Dunnett´s multiple comparisons test using GraphPad Prism v9.4.1 for Windows (GraphPad Software, www.graphpad.com). Significant p-values are indicated. # represents non-significant p-values. All p-values are available in **Supplementary Table 1**.

Vero/hACE-2/hTMPRSS2 cells were infected with the SARS-CoV-2 variants, and total and infectious viral production was measured over time. BA.1 and Gamma had the highest titers of infectious viral progeny production up to 48 hpi (**Figure 3A**). Overall, BA.1 had the highest production of viral infectious progeny (**Figure 3B**). The same pattern was observed for the total viral progeny production when the genomic RNA was quantified (**Figure 3B**). Interestingly, all variants had similar amounts of infectious virus and total viral particle production at 72 hpi (**Figures 3A, B**), suggesting that B.1.1.33 and Delta had a delayed production curve. The particle-to-PFU ratio indicates that, except for B.1.1.33 and Zeta, which had an overall higher ratio, all variants remained similar, suggesting that SARS-CoV-2 previous variants led to a higher production of defective viral particles (**Figure 3C**).

We observed that BA.1, followed by Gamma, had the highest total and infectious viral progeny production in Vero E6 cells up to 48 hpi (**Supplementary Figure 3A**). However, BA.1 titers peaked at 48 hpi and remained at 72 hpi, while Delta and B.1.1.33 peaked at 72 hpi, confirming its delayed production curve. Overall, Zeta was the least replicating variant in Vero E6 cells (**Supplementary Figures 3A, B**), correlating with an increasing particle-to-PFU ratio (**Supplementary Figure 3C**). Gamma and Delta, which achieved high viral infectious titers in 48 and 72 hpi, respectively, had the lowest particle-to-PFU ratio (**Supplementary Figure 3C**). For all these variants in both cell lines, an extensive cytopathic effect (CPE) was observed from 24 hpi up to 72 hpi. From visual inspection we could not observe any difference in the CPE extend amongst all the variants.

The results from infection of Vero E6 cells and Vero cells expressing human ACE-2 and TMPRSS2 indicate the greatest replication capacity for BA.1, followed by Gamma. Moreover, Delta replicated more efficiently than B.1.1.33 and Zeta variants, suggesting that in Vero cells, the more recent variants have a replication advantage, which, solely in the case of BA.1, in Vero-hACE cells could be explained by its greater efficiency for cell entry. The same behavior observed in Vero E6 cells highlights some advantages for these variants beyond the cell attachment, entry, and replication steps.

### 3.4 BA.1 was less infectious in a human pulmonary cell line compared to previous SARS-CoV-2 variants

**Figure 4:**
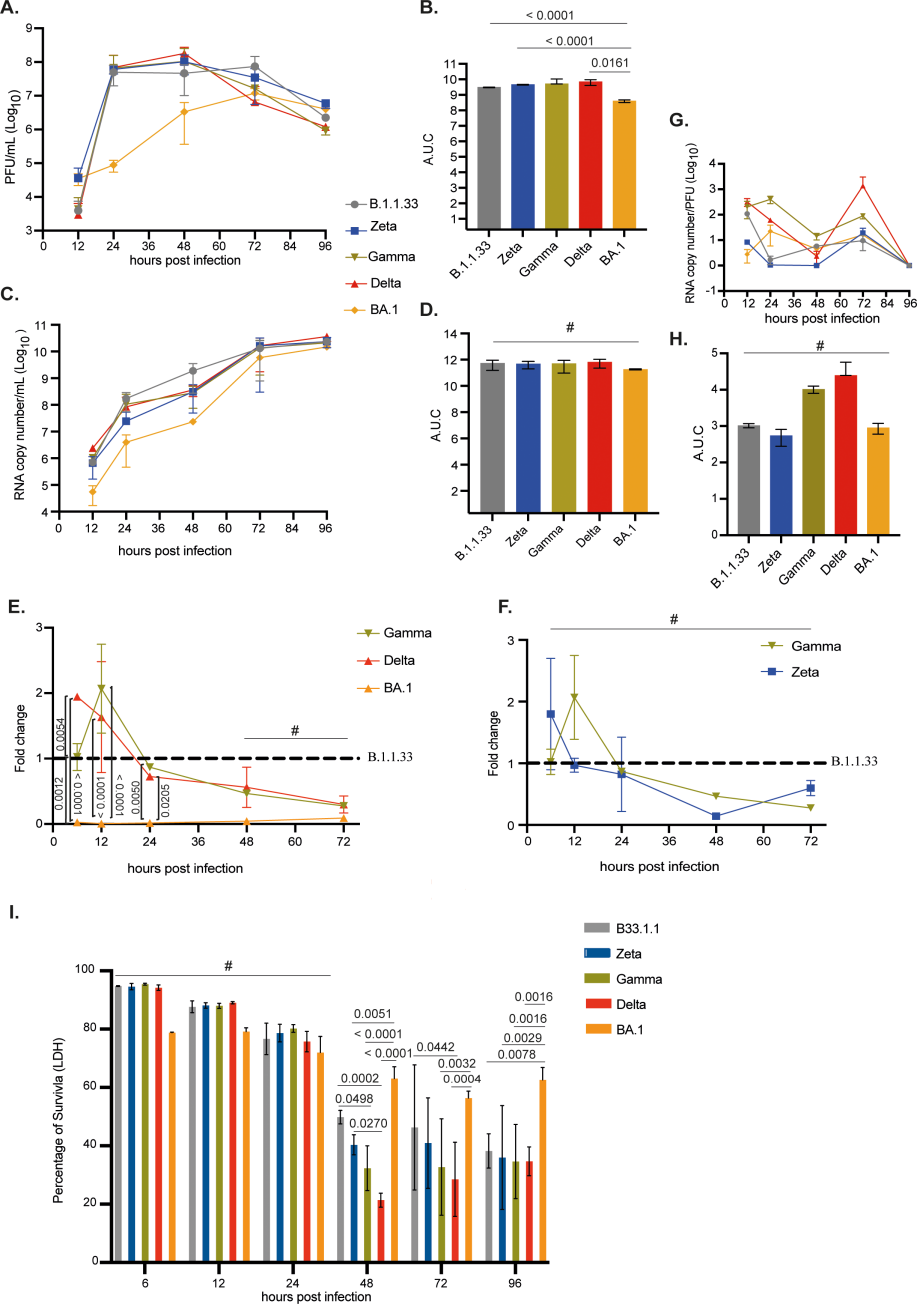
Replication of SARS-CoV-2 BA.1 variant was reduced compared to previous variants in pulmonary cells. Calu-3 cells were infected with viral variants at MOI 0.1 and incubated at 37°C with 5% CO2 for 12, 24, 48, 72, or 96 hours to evaluate viral replication kinetics. **(A)** Infectious viral progeny quantification by plaque assay at indicated time points. **(B)** Infectious viral progeny is also depicted as area under the curve (AUC). **(C)** Viral RNA copy number quantification by RT-qPCR at indicated time points. **(D)** Viral RNA copy number quantification is also depicted as AUC. **(E, F)** Nucleocapsid subgenomic RNA (sgRNA) quantification by RT-qPCR of variants Gamma, Delta, and BA.1 represented as fold change to the B.1.1.33 variant. **(D)** Nucleocapsid subgenomic RNA (sgRNA) quantification by RT-qPCR of variants Zeta and Gamma represented as fold change in relation to B.1.1.33 variant. **(G)** Particle to plaque-forming unit (P:PFU) was obtained as a measurement of viral infectivity. **(H)** P:PFU is also depicted as AUC. **(I)** Percentage of cell viability measured by LDH released in the cell culture supernatant at the indicated time points after infection. Data are shown as mean ± s.d. n=3. Statistical analyses of AUC and sgRNA quantifications were performed using one-way ANOVA followed by Dunnett´s multiple comparisons test using GraphPad Prism v9.4.1 for Windows (GraphPad Software, www.graphpad.com). Significant p-values are indicated. # represents non-significant p-values. All p-values are available in **Supplementary Table 1**.

The infection of Calu-3 cells with the SARS-CoV-2 variants showed that BA.1 had the lowest replication capacity up to 48 hpi. Levels of infectious viral progeny and total viral production were 1,000- to 15-fold and 10-fold lower, respectively, for BA.1 when compared to all other variants tested between 24–48 hpi (**Figures 4A-D**). B.1.1.33, Zeta, Gamma, and Delta presented similar infectious abilities in this cell line. Except for BA.1, after the peak of virus production at 48 hpi, the production of infectious progeny gradually decreased up to 96 hpi. AUC analysis confirmed that BA.1 had the lowest infectious virus progeny (**Figure 4B**). Levels of sgRNA, as a surrogate for RNA replication, confirmed that BA.1 replicated less during the entire course of infection (**Figure 4E**), with sgRNA levels varying from 1 to no more than 10% when compared to B.1.1.33 from 6 to 72 hpi. However, it could reach similar levels of infectious virus production to the other variants at 72 and 96 hpi, when those already show decreasing infectious titers (**Figure 4A**). Gamma and Delta had significantly higher levels of sgRNA compared to BA.1 from 6 to 24 hpi (**Figure 4E**). As for Zeta and Gamma, no differences were observed when compared to B.1.1.33 (**Figure 4F**). Overall, Gamma and Delta, the highest replicating variants in this cell line, had the highest particle-to-PFU ratio (**Figures 4G, H**). The highest replication rates of B.1.1.33, Zeta, Gamma, and Delta were associated with a significant percentage of cell-death induction from 48 hpi, especially for the Delta variant (**Figure 4I**). In contrast, for BA.1, more than 50% of cell survival was maintained up to 96 hpi, indicating that cell-induced death correlates with the extension of SARS-CoV-2 replication (**Figure 4I**). These results correlated with the CPE developed from each variant infection. Visual inspection revealed an extensive CPE from 48 hpi for all viruses, except BA.1, which gradually developed up to 96 hpi. Altogether, these results demonstrated that the BA.1 variant does not replicate efficiently in a human pulmonary cell line compared to previous SARS-CoV-2 variants.

When Calu-3-infected cells were maintained at 35°C, we still observed a lower replication rate for BA.1 compared to the other variants after 24 hpi, albeit to a lower extent at 35°C. However, the BA.1 infectious titer had a 0.5 log10 increase at 35°C when compared to 37°C, while the other variants present a statistically 2–3 log10 decrease in viral titers when replicating at 35°C (**Supplementary Figure 4**). These results indicate that lowering the temperature alleviated the disadvantage of BA.1 replication in Calu-3 cells.

### 3.5 Direct competition assay confirmed the replicative disadvantage capacity of the SARS-CoV-2 Omicron variant in Calu-3 cells but not in Vero/hACE-2/hTMPRSS2

**Figure 5:**
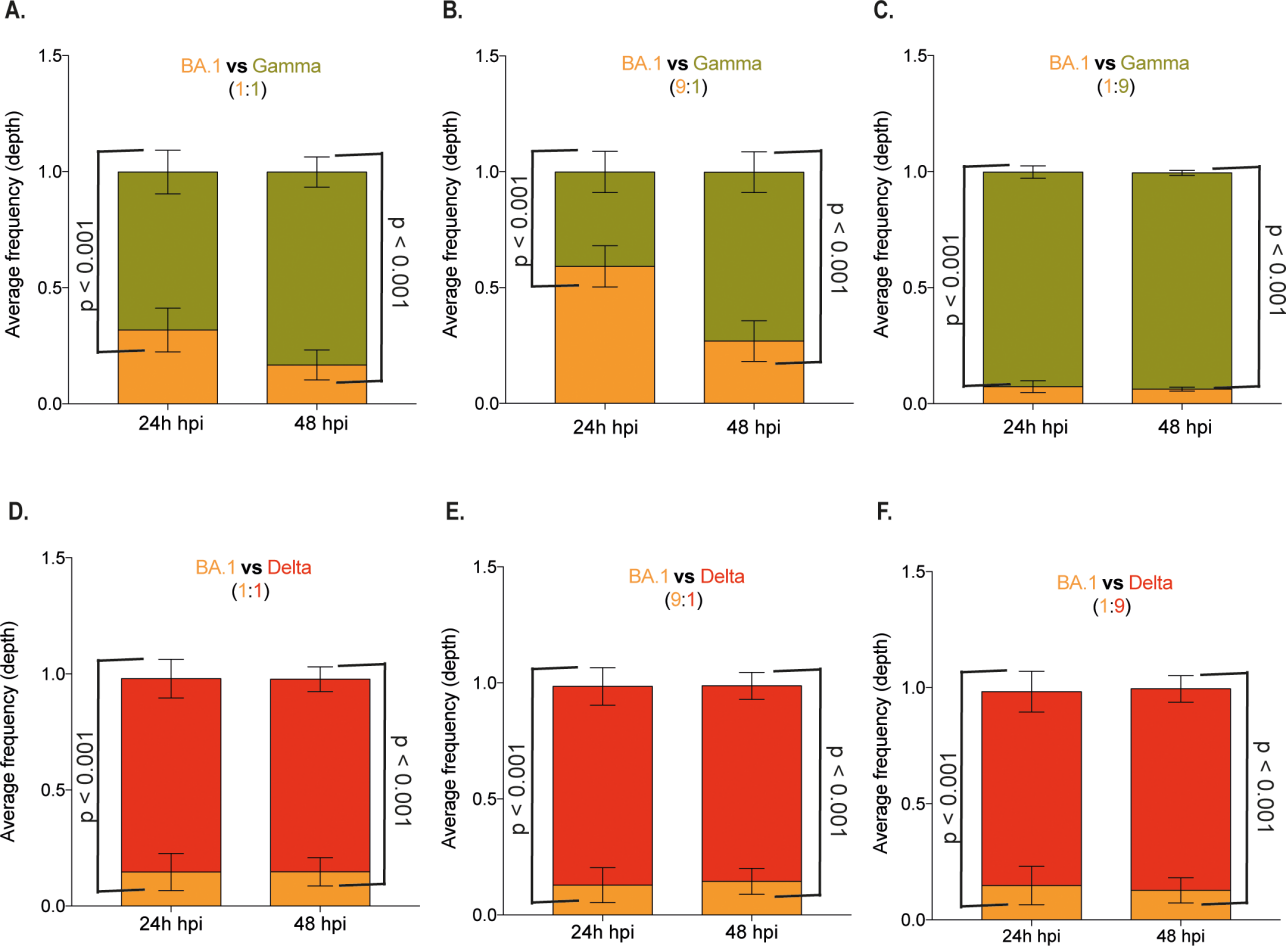
Competition assay in Calu-3 cells confirmed the lower replication of SARS-CoV-2 BA.1 variant. Calu-3 cells were co-infected with three different proportions of SARS-CoV-2 variants, resulting in a final MOI of 0.1. The infection was performed with variants Omicron BA.1 and Gamma at 1:1 **(A)**, 1:9 **(B)**, and 9:1 **(C)** proportions and with variants Omicron BA.1 and Delta at 1:1 **(D)**, 1:9 **(E)**, and 9:1 **(F)** proportions. Total RNA was extracted from culture supernatant 24- or 48-hours post- infection and subjected to NGS sequencing. The depth of each genomic region from viral lineages was plotted as the average frequency of detected sequences. Bar colors represent viral lineages BA.1 (yellow), Gamma (green), and Delta (red). Data are shown as mean ± s.d. n=3. Statistical analyses were performed using one-way ANOVA followed by Dunnett´s multiple comparisons test using GraphPad Prism v9.4.1 for Windows (GraphPad Software, www.graphpad.com). Significant p-values are indicated.

Competition assays using two SARS-CoV-2 variants to co-infect Calu-3 and Vero/hACE-2/hTMPRSS2 cells in different proportions were performed, followed by an NGS or RT-qPCR assay to detect the viral populations produced. In Calu-3 cells infected with BA.1 and Gamma, the population analysis after NGS indicated that BA.1 is the major population only when used nine times more than Gamma (9:1 proportion) at 24 hpi (**Figures 5A-C**). The Gamma variant suppressed BA.1 in all the remaining conditions. Further, Delta outgrew BA.1 even with a 9-fold higher BA.1 inoculum. In all BA.1 to Delta proportions, the frequency of the BA.1 genotype in the viral progeny population was around 10% (**Figures 5D–F**).

Co-infection in Vero/hACE-2/hTMPRSS2 cells demonstrated that Gamma only dominated over BA.1 at 24 hpi at a 1:1 proportion and at both 24 and 48 hpi at a 9-fold excess of the Gamma inoculum (**Supplementary Figures 5A–C**). While for Delta and BA.1 co-infection, BA.1 only dominated at a 9-fold excess of the BA.1 inoculum (**Supplementary Figures 5D–F**). These data confirmed the previous replication kinetics results in Vero/hACE-2/hTMPRSS2 cells, where although the BA.1 infection led to a higher infectious virus progeny production, the overall difference for Gamma and Delta was 0.5 – 1.0 log10 (**Figure 3A**). In contrast, in Calu-3 cells, BA.1 replication was markedly reduced compared to Gamma and Delta (2 log10 overall difference – **Figure 4A**).

The same assay was performed with Zeta, Gamma, and Delta variants infecting Calu-3 cells and the results were revealed by RT-qPCR. In direct competition for Gamma and Delta, the latter could not suppress Gamma replication at any proportion (**Supplementary Figures 6A–C**). At 24 hpi, even at the 9:1 Delta to Gamma proportion, only 30% of genome detection was from Delta (**Supplementary Figure 6B**). For the Zeta and Gamma competition (**Supplementary Figures 6D–F**), the Zeta variant had a replicative advantage only at the 1:9 Gamma to Zeta proportion at 24 hpi (**Supplementary Figure 6E**). Gamma outgrew Zeta in all the other conditions (**Supplementary Figure 6D**; **Supplementary Figure 5F**), demonstrating the highest fitness of Gamma compared to the previous circulating variant Zeta and the replacing Delta.

Altogether, these results suggest that viruses that became predominant in the pandemic scenario and replaced previously circulating variants do not necessarily exhibit replicative advantages in cell culture. This indicates that factors beyond virus fitness are involved in replacing a circulating SARS-CoV-2 variant with another dominant variant during the pandemic.

### 3.6 The size and number of RNA replication sites in infected cells varies amongst variants early in the SARS-CoV-2 replication cycle

**Figure 6:**
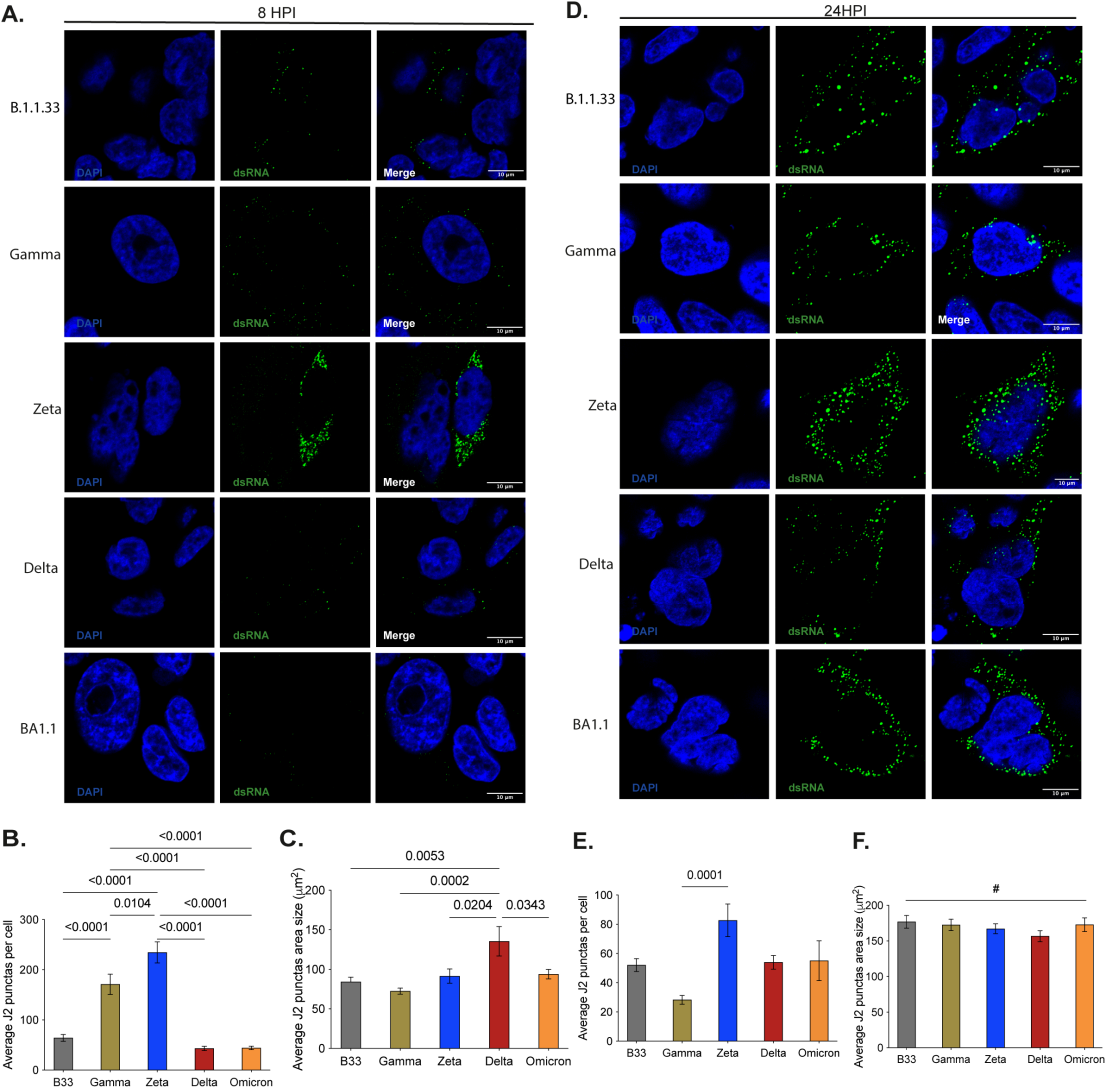
Early and late SARS-CoV-2 VOC’s replication in Calu-3 cells analysis through dsRNA immunofluorescence staining. **(A–D)** - Representative images of confocal microscopy analysis of VOC’s dsRNA accumulation at 8- and 24-hours post- infection, respectively. **(B, E)** - Average quantification data of dsRNA punctas per cell at 8- and 24-hours post-infection, respectively. To obtain the Average J2 puncta 30 random cells from 25 different fields were manually selected to generate a unique picture using the tile scan tool at ELYRA Zeiss PS.1 confocal microscope. Automate analyses, using the IMAGE J protocol, were applied to quantify total J2 puncta. Data are shown as mean ± SD. **(C, F)** - Average quantification data of dsRNA punctas area in µm2 at 8- and 24-hours post- infection. The data was obtained by Image J automatic area measurement of 181–684 cells in 25 different fields. Data are shown as mean ± SD. Statistical analyses were performed by unpaired t-test using GraphPad Prism v9.4.1 for Windows (GraphPad Software, www.graphpad.com). Significant p-values are indicated. # represents non-significant p-values. All p-values are available in **Supplementary Table 1**.

To investigate possible factors involved in the difference in variant’s fitness in tissue culture, we analyzed the early and late viral RNA replication during the viral replicative cycle in Calu-3 cells using the dsRNA as a marker. dsRNA was visualized as a predominant perinuclear puncta distribution early at infection (8 hpi) (**Figure 6A**). The average puncta per infected cell ranged from 25–250, with the lowest amount observed in Delta- and BA.1-infected cells and the highest in Zeta-infected cells (**Figure 6B**). All variants’ average puncta area size was similar, varying from 75-90μm2, except for the Delta, which had a puncta area approximately 2-fold higher (**Figure 6C**). Later during the SARS-CoV-2 replication cycle, the dsRNA puncta were readily visualized and more distributed throughout the infected cell’s cytoplasm except for BA.1, which still maintained a predominant perinuclear distribution (**Figure 6D**). At this time point, both the average of puncta per infected cell (**Figure 6E**) and the average puncta area size (**Figure 6F**) increased, except for Gamma, which had a marked reduction in the average of puncta per infected cell (**Figure 6E**). No difference was observed for the average puncta area size of Delta and the other variants at this time point (**Figure 6F**). The pattern of distribution of these puncta is similar to patterns observed for SARS-CoV-2 and other RNA-positive viruses in the earlier literature (Miller et al., 2006; Weber et al., 2006; Targett-Adams et al., 2007; Welsch et al., 2009; Son et al., 2015; Cortese et al, 2020; Lasswitz et al., 2022). Together with the readily noticeable difference in the BA.1 staining from 8 to 24 hpi, this indicates the specificity of the dsRNA staining in SARS-CoV-2-infected Calu-3 cells. Furthermore, these results demonstrate that a higher number of RNA replication sites and the size of these sites formed early in infected cells can contribute to a higher replication rate for Zeta, Gamma, and Delta, respectively. Indeed, Zeta and Delta had the highest percentage of cells actively replicating SARS-CoV-2 at 8 hpi. The increased size of the RNA replication organelle for Delta resulted in a higher rate of cells maintaining RNA replication up to 24 hpi (**Supplementary Figure 7**).

### 3.7 Spike and Nucleocapsid viral proteins accumulated to higher levels in VOC Delta Calu-3-infected cells at 24 hpi

**Figure 7:**
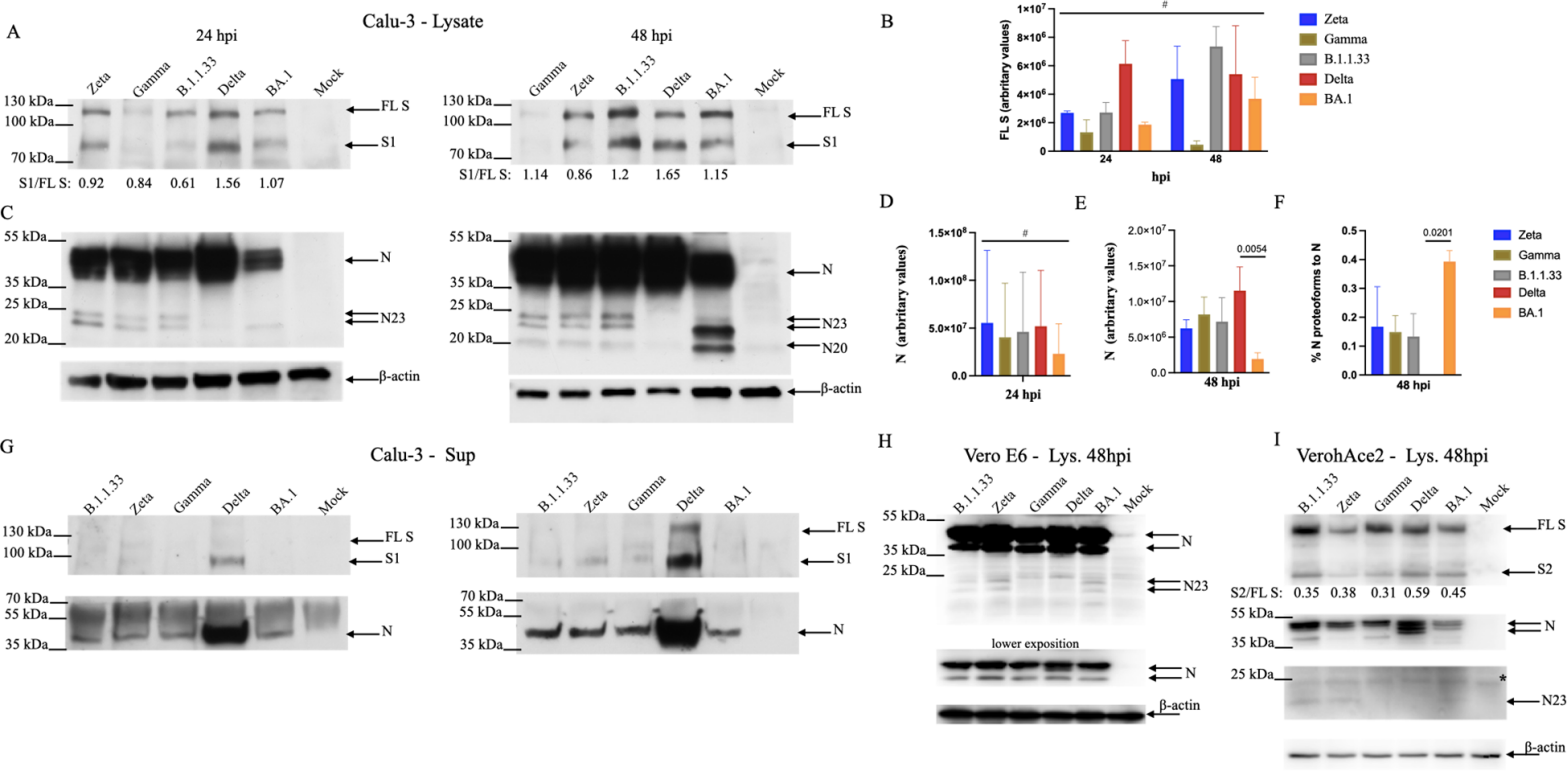
Differential levels of viral full-length and processed proteins accumulated in cells infected with SARS-CoV-2 variants. **(A)** Calu-3 cells were lysed 24- or 48 hpi and submitted to SDS-PAGE/WB with antibodies against viral S. Arrows indicated the full-length protein (FL S) and its processed S1 domain. The numbers underneath each lane represent the processing rate of FL S (S1/FL S). Data represent the average of three different experiments. **(B)** Amounts of FL S were measured by Image J and normalized by the amounts of β-actin. Data is the average of three different experiments. **(C)** SDS-PAGE/WB with antibodies against viral N protein. Arrows indicate the N protein and its proteoforms. **(D, E)** Amounts of N were measured by Image J and normalized by the amounts of β-actin at 24 and 48 hpi, respectively. Data is the average of three different experiments. The asterisk indicates p<0.05 by unpaired t-test. **(F)** The percentage of the N proteoforms to the N protein was obtained by adding up the amounts of all proteoforms observed in 48 hpi as measured by Image J and dividing it by the amount of N in 48 hpi. Data is the average of three different experiments. The asterisk indicates p<0.05 by unpaired t-test. **(G)** Cell-free supernatants submitted to SDS- PAGE/WB with antibodies against viral S and N proteins. **(H, I)** Vero E6 and Vero/hACE-2/hTMPRSS2 cells, respectively, were infected with SARS-CoV-2 variants. After 48 hours of infection, cells were lysed and submitted to SDS-PAGE/WB with antibodies against viral N protein and human β-actin, and viral S and N protein and human β-actin. The ladder marker (PageRuller Plus 10-250kDa) is shown on the left of each membrane. Statistical analyses were performed using one-way ANOVA followed by Dunnett´s multiple comparisons test using GraphPad Prism v9.4.1 for Windows (GraphPad Software, www.graphpad.com). Significant p-values are indicated. # represents non-significant p-values. All p-values are available in **Supplementary Table 1**.

We analyzed the expression levels of the main SARS-CoV-2 structural proteins, S and N, in infected Calu-3 cells at 24 and 48 hpi. All variants expressed similar levels of full-length S (FL S) at both 24 and 48 hpi (**Figures 7A, B**). As previously demonstrated, Delta had the highest S processing rate, as measured by the S1/S ratio, at both times. But, overall, all variants lead to FL S processing in the producing cell (**Figure 7A**). Spike protein levels were low in Gamma-infected cells and decreased over time (**Figures 7A, B**), which did not correlate with the high levels of infectious progeny produced at both time points (**Figure 4A**). Spike protein detection for Gamma-infected cells did not improve with longer membrane exposition (**Supplementary Figure 8**).

The N protein levels were highest in Delta-infected cells at both time points compared to BA.1 (**Figures 7C-E**), although they did not reach statistical significance at 24 hpi, showing a tendency. Three specific smaller N bands were detected in infected cells for all variants except for the Delta at 48 hpi: two of 23 kDa and one of 20 kDa, which was more apparent at 48 hpi. (**Figure 7C**). For Zeta, Gamma, and B.1.1.33 altogether, these alternative forms of N represented 12 -18% of the N protein at 48 hpi, while for BA.1, it represented on average 40% of the N protein at 48 hpi. (**Figure 7C, F**). The highest levels of structural SARS-CoV-2 proteins in Delta-infected cells reflected the highest levels of its proteins in the cell-free supernatants, with a 10- and 5-fold excess for S and N, respectively when compared to Zeta, Gamma, and B.1.1.33 and 12- and 7-fold excess for S and N, respectively, when compared to BA.1 (**Figure 7G**). For the S glycoprotein, we predominantly detected the processed S1 domain, while the extra N forms detected in the infected cells’ lysates were not observed in the cell-free supernatants.

These results demonstrate that the differences observed in infectious virus production in BA.1-Calu-3 cells could be partially explained by the different levels of viral structural protein synthesis. Analyzing the N protein content in Vero E6 and Vero-hACE2/hTMPRSS2 infected cells, in which these variants replicate at equivalent efficiencies (**Figures 4A**; **6**), we did not observe substantial differences (**Figures 7H, I**). The same 23 kDa smaller N bands detected in Calu-3 infected cells were also detected in Vero E6, albeit in lower levels for BA.1 (**Figure 7H**), while in Vero-hACE2/hTMPRSS2 only a faint band of 23 kDa was observed (**Figure 7I**). FL S processing was also observed for all variants in Vero-hACE2/hTMPRSS2 cells (**Figure 7I**).

## 4 Discussion

The emergence of viral variants, such as Alpha, Beta, Gamma, Delta, and Omicron BA.1, during the COVID-19 pandemic represented a significant threat to the global human population. The high increase of morbidity/mortality with the emergence of variants like Alpha, Gamma, and Delta, in the pre-vaccination scenario, and the unprecedented wave of BA.1 already with an important number of two-dose vaccinated world population demonstrated the lower sensitivity of these variants to neutralizing sera from previously infected or post-vaccinated individuals (Halfmann et al., 2022; Peacock et al., 2022; Laitman et al., 2022; Sentis et al., 2022; de Souza et al., 2021a; Lopez Bernal et al., 2021; Evans et al., 2021; Zeng et al., 2021; Carreño et al., 2021; Diamond et al., 2021; de Souza et al., 2021b) establishing the host’s immune response as the driving force in shaping SARS´CoV-2 evolution In this scenario, the newly acquired mutations in the emerging variants have the potential to modify its replication capacity or biological characteristics benefiting the virus to better adapt to the host or reducing its fitness. Since SARS-CoV-2 continues to evolve, a comprehensive study of previous variants’ replication characteristics will help to establish viral features guiding virus adaptation to be readily detected in the future, allowing a better assessment of the risk of the emerging variants. Thus, this study characterized the replication capacity of SARS-CoV-2 variants that circulated during the COVID-19 pandemic, including an ancient D614G virus (B.1.1.33), and the viral variants Zeta, Gamma, Delta, and BA.1, that sequentially replaced each other in the population. We demonstrated that the replication capacity of these variants depended on the cell type; for instance, Zeta, Gamma, and Delta replicated better in a human lung epithelial cell line. BA.1 instead replicated to the highest titers in macaque epithelial kidney cells, independently of the TMPRSS2 presence. It has been demonstrated that BA.1 preferentially uses the endocytic entry route and is less sensitive to TMPRSS2 inhibitors (Begum et al., 2024; Willett et al., 2022; Meng et al., 2022). Indeed, in our virus entry assays with native viruses, we observed a clear advantage for BA.1 in Vero-hACE2/hTMPRSS2 cells, possibly reflecting its more efficient use of the endocytic machinery. Previous studies reported a rate-limiting intermediate in the HIV fusion process that can be arrested by decreasing temperature (Melikyan et al., 2000; Henderson and Hope, 2006). If the S protein binding to its receptor is not rate-limiting, lowering the temperature could favor endocytosis by reducing the fusion constant (Chou, 2007). Thus, in our assay, the binding temperature (4°C) could have affected the S fusion dynamics with the cell membrane, favoring endocytosis and yielding BA.1 an advantage over the previous variants, more prone to cell-fusion entry.

In direct competition assays, BA.1 was fitter than Gamma to replicate in Vero-hACE2/hTMPRSS2 cells up to 48 hpi, although Gamma prevailed at 24 hpi, thus reflecting the single-infection kinetics in this cell line. These results can be attributed to the higher efficiency of BA.1 entry into these cells. However, the same pattern of single-infection kinetics was observed in Vero E6 cells, in which BA.1 and Gamma surface membrane attachment and cell entry were equivalent. Thus, other viral characteristics besides binding and entry could explain this replicative difference. On the other hand, although total and infectious virus titers were significantly higher for BA.1 than for Delta in Vero-hACE2/hTMPRSS2 cells, Delta was fitter to replicate in this cell line as indicated by the direct competition assays. These data suggest that viral factors beyond the early steps of virus entry affect the replication efficiency of these variants. Indeed, it has been demonstrated that S properties, such as binding to ACE2 and fusogenicity, do not explain differences in replication amongst SARS-CoV-2 variants (Begum et al., 2024). However, Meng et al. (2022) suggested that Omicron entry is impaired in cell lines that highly express TMPRSS2, which probably explains the lower replicative capacity of this variant in native virus replication assays using various cell lines. In contrast, our data showed no significant differences among all variants in cell attachment and entry in Calu-3 cells, nor did they reveal an advantage of BA.1 entry in Vero-hACE2/hTMPRSS2. These discrepancies could potentially result from limitations of the artificial system employed by Meng et al. (2022) to directly access virus entry, mostly related to an envelope composition, lacking the main viral membrane protein, and to a different nucleocapsid composition that imposes different interactions to the S transmembrane portion.

Previous studies reported, *in vitro* and *in vivo*, differences in the replication capacity of ancestral SARS-CoV-2 D614G when compared to different viral variants (Hui et al., 2022a; Peacock et al., 2022; Hui et al., 2022b; Begum et al., 2024; Meng et al., 2022; Nchioua et al., 2023; Mautner et al., 2022). Overall, BA.1 replicates to lower titers in epithelial cells of the lung and intestine, primary lung cells, and *ex vivo* tissue. There are some heterogeneities when comparing ancestral D614G, Alpha, Beta, Gamma, and Delta replication capacity, but higher replication titers for Alpha, Gamma, and Delta have been reported (Hui et al., 2022b; Meng et al., 2022; Nchioua et al., 2023; Mautner et al., 2022). Besides evidence suggesting that the attachment/entry steps correlate with these differences, viral factors that could contribute to the replicative characteristics of the SARS-CoV-2 variants are lacking. Nchioua et al. (2023) demonstrated that BA.1 is more resistant to Interferons than previous viruses when replicating in both Calu-3 and iPSC-derived iAT-2 cells. A cell-dependent phenomenon is evident since SARS-CoV-2 replication in bronchial cells *ex vivo* (Hui et al., 2022a) and in hamster upper respiratory tract (Escalera et al., 2022) favors BA.1. Another study by Hui et al (2022b) demonstrated that BA.1 presents different replicative kinetics according to the temperature of incubation and the cells from the upper and lower respiratory tract. Since the physiological temperature of the mammal’s upper respiratory tract varies between 33-35°C, we reproduced this condition by incubating infected Calu-3 cells at 35°C. The fact that BA.1 was insensitive to temperature change while ancestral D614G, Zeta, Gamma, and Delta had an average 2log10 drop in infectious titers suggests a role for virus-cell interactions to explain BA.1 replicative advantage in the upper respiratory tract (Stauft et al., 2023). Virus adaptation to replicate at lower temperatures has been demonstrated for Influenza A. For instance, avian Influenza A with a PB2 polymerase mutation could efficiently replicate in mammalian cells when the temperature was reduced (Hayashi et al., 2015). In contrast, a Nucleoprotein mutation at a conserved phosphorylation site in another Influenza A isolate resulted in a cold-sensitivity phenotype (Zheng et al., 2021). This phenomenon observed in Influenza could explain why the differences in Zeta, Gamma, Delta, and BA.1 replicative capacity may be related to viral RNA replication and assembly.

The lower infectious progeny production from BA.1-infected Calu-3 cells could be due to an inefficient accumulation of sgRNAs, especially at 6–12 hpi, which coincided with the lowest average number of dsRNA puncta in BA.1-infected cells and the lowest number of cells positive for dsRNA at 8 hpi. The fittest variants in Calu-3 cells – Gamma, Delta and Zeta – were the ones that had the highest levels of sgRNA from 6 to 12 hpi., the highest average number of dsRNA puncta per cell (Gamma and Zeta), and also the highest percentage of dsRNA positive cells (Zeta and Delta) at 8 hpi.

The double-membrane vesicles (DMVs), which serve as sites for SARS-CoV-2 viral RNA replication, are formed by the association of the viral Nsp3 and Nsp4 with the Endoplasmic Reticulum (ER) and the induction of ER membrane curvature, thereby generating these RNA replication organelles (ROs). Cryo-tomography analyses showed that Nsp3 and Nsp4 are sufficient to form a pore in the ROs, which will serve to translocate the newly synthesized viral genomic RNA and the sgRNAs from the DMV lumen to the cytoplasm (Klein et al., 2020; Zimmermann et al., 2023) to be assembled into new ribonucleocapsid structures, and to be translated, respectively (Zimmermann et al., 2023). Cryo-tomography analyses also characterized the inner average diameter of the DMV as 338 nm (Klein et al., 2020), which implies an average area of 89.68 μm^2^. Indeed, we observe equivalent puncta area average for all variants, except for Delta, which had an average size twice as high as the other variants. The data demonstrated that variants inducing higher DMVs average number and size early on infection accumulate higher levels of sgRNA. Although dsRNA staining of uninfected cells with the J2 antibody can be detected as a weak signal, it has been widely demonstrated that RNA-positive infected cells present a strong cytoplasmic signal upon J2 staining (Weber et al., 2006). Several other studies have demonstrated early specific perinuclear dsRNA staining of cells infected with Dengue virus, Hepatitis C virus, Chikungunya virus, and SARS-CoV-2 (Miller et al., 2006; Cloherty et al., 2024; Targett-Adams et al., 2007; Lasswitz et al., 2022; Cortese et al., 2020). The early appearance, size, and distribution of the dsRNA staining in our study correlate with those demonstrated by Cortese and coworkers (Cortese et al., 2020). Calu-3 cells are highly permissive to SARS-CoV-2 infection. A specific, strong dsRNA signal, distinct from the signal from uninfected cells, was readily observed at 8 hpi (**Figure 6A**), while it has been demonstrated that dsRNA-specific staining can be detected in SARS-CoV-2-infected Calu-3 cells as early as 6 hpi (Cortese et al., 2020). At 24 hpi (**Figure 6B**), a stronger dsRNA signal undoubtedly distinguished infected from uninfected Calu-3 cells. Taken together, these observations allowed us to conclude that the dsRNA staining observed in SARS-CoV-2-infected Calu-3 cells was specific.

Our data shows a positive correlation of higher levels of viral RNA replication with higher levels of SARS-CoV-2 structural N protein in infected cells. Moreover, not only did N protein levels vary between BA.1 and all other variants but also the pronounced accumulation of 25-22kDa smaller bands in Calu-3 cells. The appearance of smaller size proteoforms of SARS-CoV-2 N occurs upon its heterologous expression in various systems (Meyer et al., 2021; Emmott et al., 2013) and has been related to cleavage by cellular Caspases (Diemer et al., 2008). However, a detailed work demonstrated that N purified from *E. coli* without contamination with cellular proteases underwent “cleavage” generating smaller size proteins of 29 – 25kDa (Mark et al., 2008). These authors suggested an unusual lability of N. Also, two works analyzing the SARS- CoV-2 N protein by native mass spectrometry demonstrated the presence of these smaller proteoforms from purified protein expressed in *E. coli* (Lutomski et al., 2021), and from SARS-CoV-2 infected cells (Meyer et al., 2021), with most of the proteoforms coinciding in both studies. The full-length N has amino acids and 45.8kDa, Lutomski (Lutomski et al., 2021), demonstrated the existence of 5 proteoforms; 4 with a C-terminal cleavage/breakage (N_1-392_ – 42.9kDa, N_1-273_ – 29.4kDa, N_1-220_ – 23.5kDa, N_1-209_ –22.5kDa), and 1 with a N-terminal cleavage/breakage (N_156-419_ – 28.7kDa) (**Supplementary Figure 9**). This unexpected lability of N can be explained by the presence of a protein domain rich in the serine-arginine (SR) dipeptide sequence (N-SR-rich domain) which is statistically more likely to be present in proteins with shorter than longer *in vivo* half-lives (Mark et al., 2008; Guruprasad et al., 1990). Thus, the SR-rich domain makes N “unstable”. A high degree of conservation amongst all variants was observed at the proposed cleavage/breakage sites. However, for Delta, the R203M and the G215C substitutions, just preceding the N220 and N273 cleavage/breakage, respectively, close to the N-SR-rich domain, could explain the almost absence of N proteoforms during Delta infection.

Interestingly, except for Delta, Beta, and previous related variants of interest (VOIs), all variants derived from the ancestral D614G fixed the RG203/204KR substitution near the N-SR-rich domain, which has been associated with an increase in N phosphorylation compared to the original RG sequence (Johnson et al., 2022). When we inspected the N expression in Calu-3 cells infected with a Wuhan-related SARS-CoV-2 A2 isolate, we observed the presence of a small amount of the single proteoform of 23kDa (**Supplementary Figure 8**), suggesting that the KR substitution could contribute to an additional degree of instability of N. Although all the C-terminal proteoforms can bind to the viral genomic RNA a disequilibrium could be detrimental for virus replication, since only dimmers of the full-length protein can bind to the viral genomic RNA for assembly (Lutomski et al., 2021). For instance, N and Nsp3 interaction at the sites of viral RNA replication is necessary for process efficiency (Li et al., 2023). The presence of N could account for ROs maintenance and efficiently sort out the RNA species being translocated from the interior of the DMVs to sites of virus assembly. Thus, the apparent greater instability of the full-length N during the BA.1 replication could explain its reduced ability to replicate in Calu-3 cells. In addition, small amounts of the N proteoforms were observed in Vero E6 and Vero-hACE2/hTMPRSS2 cells for all the variants, including BA.1, which replicate efficiently in this cell line. As demonstrated for the Nucleoprotein mutation at a conserved phosphorylation site in Influenza A, which reduced protein oligomerization leading to a cold-sensitivity phenotype (Zheng et al., 2021), the high sensitivity of B.1.133, Zeta, Gamma, and Delta to lower virus replication temperature in Calu-3 could be related to greater N instability due to its supposed less efficient oligomerization, which would not affect more BA.1. BA.1 Nsp3 has unique substitutions in functional relevant domains that could potentially affect its ability to bind N (Hossain et al., 2022), while the N mutations P13L and ΔERS31–33 residing in the N-arm domain preceding the N-terminal domain (**Supplementary Figure 9**), which has an RNA binding modulatory role could affect the RNA binding regulation by N. Together it could contribute to a greater N instability.

The determinants of successive selection of SARS-CoV-2 variants are related to a myriad of factors, including escaping from immune response elicited by vaccination and/or natural infection, higher transmissibility rates, and intrinsic viral factors. The contribution of intrinsic viral factors is directly related to differences observed in virus replicative capacity among variants, and their characterization will contribute to a broader understanding of the epidemic properties of the following emerging SARS-CoV-2 variants. In this sense, this work contributes to demonstrating the importance of the RNA replication step and the role of N in shaping the replicative characteristics of the SARS-CoV-2 variants.

## Data Availability

The datasets presented in this study can be found in online repositories. The names of the repository/repositories and accession number(s) can be found below: https://gisaid.org/, EPI_ISL_11836071 EPI_ISL_18433588 EPI_ISL_18433589 EPI_ISL_18433882 EPI_ISL_18433590 EPI_ISL_18433591 EPI_ISL_18433883 EPI_ISL_18433592 EPI_ISL_7699344 EPI_ISL_18433884 EPI_ISL_18433885 EPI_ISL_18433886 EPI_ISL_18433887.

## References

[B1] Aksamentov I. Roemer C. Hodcroft E. B. Neher R. A. (2021). Nextclade: clade assignment, mutation calling and quality control for viral genomes. J. Open Source Software 6, 3773. doi: 10.21105/joss.03773

[B2] Banete A. Griffin B. D. Corredor J. C. Chien E. Yip L. Gunawardena T. N. A.. (2025). Pathogenesis and transmission of SARS-CoV-2 D614G, Alpha, Gamma, Delta, and Omicron variants in golden hamsters. NPJ Viruses 3(1), 15. doi: 10.1038/s44298-025-00092-2, PMID: 40295859 PMC11850601

[B3] Begum M. M. Ichihara K. Takahashi O. Nasser H. Jonathan M. Tokunaga K.. (2024). Virological characteristics correlating with SARS-CoV-2 spike protein fusogenicity. Front. Virol. 4. doi: 10.3389/fviro.2024.1353661

[B4] Campbell F. Archer B. Laurenson-Schafer H. Jinnai Y. Konings F. Batra N.. (2021). Increased transmissibility and global spread of SARS-CoV-2 variants of concern as at June 2021. Eurosurveillance 26 (24). doi: 10.2807/1560-7917.es.2021.26.24.2100509, PMID: 34142653 PMC8212592

[B5] Candido D. S. Claro I. M. de Jesus J. G. Souza W. M. Moreira F. R. R. Dellicour S.. (2020). Evolution and epidemic spread of SARS-CoV-2 in Brazil. Science 369, 1255–1260. doi: 10.1126/science.abd2161, PMID: 32703910 PMC7402630

[B6] Carreño J. M. Alshammary H. Tcheou J. Singh G. Raskin A. Kawabata H.. (2021). Activity of convalescent and vaccine serum against SARS-CoV-2 Omicron. Nature 602 (7898), 682–688. doi: 10.1038/s41586-022-04399-5, PMID: 35016197

[B7] Chou T. (2007). Stochastic entry of enveloped viruses: fusion versus endocytosis. Biophys. J. 93, 1116–1123. doi: 10.1529/biophysj.107.106708, PMID: 17513379 PMC1929032

[B8] Cloherty A. P. M. Rader A. G. Patel K. S. Eisden T. T. H. D. Van Piggelen S. Schreurs R. R. C. E.. (2024). Dengue virus exploits autophagy vesicles and secretory pathways to promote transmission by human dendritic cells. Front. Immunol. 15. doi: 10.3389/fimmu.2024.1260439, PMID: 38863700 PMC11165123

[B9] Cortese M. Lee J. Cerikan B. Neufeldt C. J. Oorschot V. M. Köhrer S.. (2020). Integrative imaging reveals SARS-COV-2-Induced reshaping of subcellular morphologies. Cell Host Microbe 28, 853–866.e5. doi: 10.1016/j.chom.2020.11.003, PMID: 33245857 PMC7670925

[B10] de Souza W. M. Amorim M. R. Sesti-Costa R. Coimbra L. D. Brunetti N. S. Toledo-Teixeira D. A.. (2021b). Neutralisation of SARS-CoV-2 lineage P.1 by antibodies elicited through natural SARS-CoV-2 infection or vaccination with an inactivated SARS-CoV-2 vaccine: an immunological study. Lancet Microbe. 2 (10), e527–e535. doi: 10.1016/s2666-5247(21)00129-4, PMID: 34258603 PMC8266272

[B11] de Souza W. M. Muraro S. P. Souza G. F. Amorim M. R. Sesti-Costa R. Mofatto L. S.. (2021a). Clusters of SARS-coV-2 lineage B.1.1.7 infection after vaccination with adenovirus-vectored and inactivated vaccines. Viruses 2 (10), e527–e535. doi: 10.3390/v13112127, PMID: 34834934 PMC8623206

[B12] Dezordi F. Z. Da Silva Neto A. M. De Lima Campos T. Jerônimo P. M. C. Aksenen C. F. De Almeida S. C.. (2022). ViralFlow: a versatile automated workflow for SARS-COV-2 genome assembly, lineage assignment, mutations and intrahost variant detection. Viruses 14, 217. doi: 10.3390/v14020217, PMID: 35215811 PMC8877152

[B13] Dhama K. Nainu F. Frediansyah A. Yatoo M. I. Mohapatra R. K. Chakraborty S.. (2023). Global emerging Omicron variant of SARS-CoV-2: Impacts, challenges and strategies. J. Infection Public Health 16, 4–14. doi: 10.1016/j.jiph.2022.11.024, PMID: 36446204 PMC9675435

[B14] Diamond M. P. Rita P.-Y. Xie X. Case J. F. Zhang X. VanBlargan L. A.. (2021). SARS-CoV-2 variants show resistance to neutralization by many monoclonal and serum-derived polyclonal antibodies. Res. Square. Available at: https://www.researchsquare.com/article/rs-228079/v1. 10.1038/s41591-021-01294-w PMC8058618 33664494

[B15] Diemer C. Schneider M. Seebach J. Quaas J. Frösner G. Schätzl H. M.. (2008). Cell type-specific cleavage of nucleocapsid protein by effector caspases during SARS coronavirus infection. J. Mol. Biol. 376, 23–34. doi: 10.1016/j.jmb.2007.11.081, PMID: 18155731 PMC7094231

[B16] Emmott E. Munday D. Bickerton E. Britton P. Rodgers M. A. Whitehouse A.. (2013). The cellular interactome of the coronavirus infectious bronchitis virus nucleocapsid protein and functional implications for virus biology. J. Virol. 87, 9486–9500. doi: 10.1128/JVI.00321-13, PMID: 23637410 PMC3754094

[B17] Escalera A. Gonzalez-Reiche A. S. Aslam S. Mena I. Laporte M. Pearl R. L.. (2022). Mutations in SARS-CoV-2 variants of concern link to increased spike cleavage and virus transmission. Cell Host Microbe 30, 373–387.e7. doi: 10.1016/j.chom.2022.01.006, PMID: 35150638 PMC8776496

[B18] Evans J. P. Zeng C. Carlin C. Lozanski G. Saif L. J. Oltz E. M.. (2021). Loss of Neutralizing Antibody Response to mRNA Vaccination against SARS-CoV-2 Variants: Differing Kinetics and Strong Boosting by Breakthrough Infection. bioRxiv. 2021.12.06.471455. doi: 10.1101/2021.12.06.471455v1, PMID: 34909777

[B19] Faria N. R. Claro I. M. Candido D. D. Franco L. A. Andrade S. Coletti T. M.. (2021). Genomic characterization of an emergent SARS-CoV-2 lineage in Manaus: preliminary findings. Available online at: https://virological.org/t/genomic-characterisation-of-an-emergent-sars-cov-2-lineage-in-manaus-preliminary-findings/586 (Accessed December 17, 2024).

[B20] Genomahcov-F. (2021). Dashboard-en. Available online at: https://www.genomahcov.fiocruz.br/dashboard-en/ (Accessed February 20, 2025).

[B21] GISAID - gisaid.org (GISAID). Available online at: https://gisaid.org/ (Accessed May 14, 2024).

[B22] Gupta S. Gupta D. Bhatnagar S. (2024). Analysis of SARS-CoV-2 genome evolutionary patterns. Microbiol. spectrum. 12 (2), e0265423. doi: 10.1128/spectrum.02654-23, PMID: 38197644 PMC10846092

[B23] Guruprasad K. Reddy B. Bhasker. V. Pandit M. W. (1990). Correlation between stability of a protein and its dipeptide composition: a novel approach for predicting *in vivo* stability of a protein from its primary sequence. Protein Engineering Design Selection 4, 155–161. doi: 10.1093/protein/4.2.155, PMID: 2075190

[B24] Halfmann P. J. Iida S. Iwatsuki-Horimoto K. Maemura T. Kiso M. Scheaffer S. M.. (2022). SARS-CoV-2 Omicron virus causes attenuated disease in mice and hamsters. Nature 603, 687–692. doi: 10.1038/s41586-022-04441-6, PMID: 35062015 PMC8942849

[B25] Hayashi T. Wills S. Bussey K. A. Takimoto T. (2015). Identification of influenza A virus PB2 residues involved in enhanced polymerase activity and virus growth in mammalian cells at low temperatures. J. Virol. 89, 8042–8049. doi: 10.1128/jvi.00901-15, PMID: 26018156 PMC4505657

[B26] Hénaut M. Carbonneau J. Rhéaume C. Levade I. Boivin G. (2023). *In vitro* fitness of SARS-CoV-2 variants as assessed by competition experiments followed by ddRT-PCR and whole genome sequencing. J. Clin. Virol. 165, 105517. doi: 10.1016/j.jcv.2023.105517, PMID: 37321149 PMC10245455

[B27] Henderson H. I. Hope T. J. (2006). The temperature arrested intermediate of virus-cell fusion is a functional step in HIV infection Virol. J. 3, 36. doi: 10.1186/1743-422x-3-36, PMID: 16725045 PMC1482684

[B28] Hodcroft E. B. (2021). CoVariants: SARS-coV-2 mutations and variants of interest. Available online at: https://covariants.org/cases (Accessed April 18, 2021).

[B29] Hossain A. Akter S. Rashid A. A. Khair S. Alam A.S.M.R.U. (2022). Unique mutations in SARS-CoV-2 Omicron subvariants’ non-spike proteins: Potential impacts on viral pathogenesis and host immune evasion. Microbial Pathogenesis 170, 105699. doi: 10.1016/j.micpath.2022.105699, PMID: 35944840 PMC9356572

[B30] Hou Y. J. Chiba S. Halfmann P. Ehre C. Kuroda M. Dinnon K. H.. (2020). SARS-CoV-2 D614G Variant Exhibits Enhanced Replication ex vivo and Earlier Transmission in *vivo* . bioRxiv. 2020.09.28.317685. doi: 10.1101/2020.09.28.317685, PMID: 33184236 PMC7775736

[B31] Hui K. P. Y. Ho J. C. W. Cheung M. Ng K. Ching R. H. H. Lai K.. (2022a). SARS-CoV-2 Omicron variant replication in human bronchus and lung ex vivo. Nature. 603 (7902), 715–720. doi: 10.1038/s41586-022-04479-6, PMID: 35104836

[B32] Hui K. P. Y. Ng K.-C. Ho J. C. W. Yeung H.-W. Ching R. H. H. Gu H.. (2022b). Replication of SARS-CoV-2 Omicron BA.2 variant in ex vivo cultures of the human upper and lower respiratory tract. EBioMedicine 83, 104232. doi: 10.1016/j.ebiom.2022.104232, PMID: 35988466 PMC9387351

[B33] Johnson B. A. Zhou Y. Lokugamage K. G. Vu M. N. Bopp N. Crocquet-Valdes P. A.. (2022). Nucleocapsid mutations in SARS-CoV-2 augment replication and pathogenesis. PLOS. Pathogens 18, e1010627. doi: 10.1371/journal.ppat.1010627, PMID: 35728038 PMC9275689

[B34] Klein S. Cortese M. Winter S. L. Wachsmuth-Melm M. Neufeldt C. J. Cerikan B.. (2020). SARS-CoV-2 structure and replication characterized by in *situ* cryo-electron tomography. Nat. Commun. 11 (1). doi: 10.1038/s41467-020-19619-7, PMID: 33208793 PMC7676268

[B35] Korber B. Fischer W. M. Gnanakaran S. Yoon H. Theiler J. Abfalterer W.. (2020). Tracking changes in SARS-coV-2 spike: evidence that D614G increases infectivity of the COVID-19 virus. Cell. 182 (4). doi: 10.1016/j.cell.2020.06.043, PMID: 32697968 PMC7332439

[B36] Laitman A. M. Lieberman J. A. Hoffman N. G. Roychoudhury P. Mathias P. C. Greninger A. L. (2022). The SARS-coV-2 omicron variant does not have higher nasal viral loads compared to the delta variant in symptomatic and asymptomatic individuals. J. Clin. Microbiol. 60 (4), e0013922. doi: 10.1128/jcm.00139-22, PMID: 35341324 PMC9020334

[B37] Lasswitz L. Zapatero-Belinchón F. J. Moeller R. Hülskötter K. Laurent T. Carlson L.. (2022). The tetraspanin CD81 is a host factor for chikungunya virus replication. mBio 13 (3). doi: 10.1128/mbio.00731-22, PMID: 35612284 PMC9239085

[B38] Li B. Deng A. Li K. Hu Y. Li Z. Shi Y.. (2022). Viral infection and transmission in a large, well-traced outbreak caused by the SARS-CoV-2 Delta variant. Nat. Commun. 13, 460. doi: 10.1038/s41467-022-28089-y, PMID: 35075154 PMC8786931

[B39] Li P. Xue B. Schnicker N. J. Wong L.-Y. R. Meyerholz D. K. Perlman S. (2023). Nsp3-N interactions are critical for SARS-CoV-2 fitness and virulence. Proc. Natl. Acad. Sci. United States America 120, e2305674120. doi: 10.1073/pnas.2305674120, PMID: 37487098 PMC10400999

[B40] Lopez Bernal J. Andrews N. Gower C. Gallagher E. Simmons R. Thelwall S.. (2021). Effectiveness of covid-19 vaccines against the B.1.617.2 (Delta) variant. New Engl. J. Med. 385 (7). doi: 10.1056/nejmoa2108891, PMID: 34289274 PMC8314739

[B41] Lutomski C. A. El-Baba T. J. Bolla J. R. Robinson C. V. (2021). Multiple roles of SARS-coV-2 N protein facilitated by proteoform-specific interactions with RNA, host proteins, and convalescent antibodies. JACS Au 1, 1147–1157. doi: 10.1021/jacsau.1c00139, PMID: 34462738 PMC8231660

[B42] Mark J. Li X. Cyr T. Fournier S. Jaentschke B. Hefford M. A. (2008). SARS coronavirus: Unusual lability of the nucleocapsid protein. Biochem. Biophys. Res. Commun. 377, .429–.433. doi: 10.1016/j.bbrc.2008.09.153, PMID: 18926799 PMC7092863

[B43] Mautner L. Hoyos M. Dangel A. Berger C. Ehrhardt A. Baiker A. (2022). Replication kinetics and infectivity of SARS-CoV-2 variants of concern in common cell culture models. Virol. J. 19 (1). doi: 10.1186/s12985-022-01802-5, PMID: 35473640 PMC9038516

[B44] Melikyan G. B. Markosyan R. M. Hemmati H. Delmedico M. K. Lambert D. M. Cohen F. S. (2000). Evidence that the transition of HIV-1 gp41 into a six-helix bundle, not the bundle configuration, induces membrane fusion. J. Cell Biol. 151, 413–424. doi: 10.1083/jcb.151.2.413, PMID: 11038187 PMC2192659

[B45] Meng B. Abdullahi A. Ferreira I. A. T. M. Goonawardane N. Saito A. Kimura I.. (2022). Altered TMPRSS2 usage by SARS-CoV-2 Omicron impacts tropism and fusogenicity. Nature. 603 (7902), 706–714. doi: 10.1038/s41586-022-04474-x, PMID: 35104837 PMC8942856

[B46] Meyer B. Chiaravalli J. Gellenoncourt S. Brownridge P. Bryne D. P. Daly L. A.. (2021). Characterising proteolysis during SARS-CoV-2 infection identifies viral cleavage sites and cellular targets with therapeutic potential. Nat. Commun. 12, 5553. doi: 10.1038/s41467-021-25796-w, PMID: 34548480 PMC8455558

[B47] Miller S. Sparacio S. Bartenschlager R. (2006). Subcellular localization and membrane topology of the dengue virus type 2 non-structural protein 4B. J. Biol. Chem. 281, 8854–8863. doi: 10.1074/jbc.m512697200, PMID: 16436383

[B48] Moreira F. R. R. D’arc M. Mariani D. Herlinger A. L. Schiffler F. B. Rossi Á.D.. (2021). Epidemiological dynamics of SARS-CoV-2 VOC Gamma in Rio de Janeiro, Brazil. Virus Evol. 7 (2). doi: 10.1093/ve/veab087, PMID: 34725568 PMC8522364

[B49] Naveca F. G. Nascimento V. de Souza V. C. Corado A. de L. Nascimento F.. (2021a). COVID-19 in Amazonas, Brazil, was driven by the persistence of endemic lineages and P.1 emergence. Nat. Med. 27, 1230–1238. doi: 10.1038/s41591-021-01378-7, PMID: 34035535

[B50] Naveca F. G. Nascimento V. Souza V. Corado A. Nascimento F. Silva G.. (2021b). Phylogenetic relationship of SARS-CoV-2 sequences from Amazonas with emerging Brazilian variants harboring mutations E484K and N501Y in the Spike protein. Available online at: https://virological.org/t/phylogenetic-relationship-of-sars-cov-2-sequences-from-amazonas-with-emerging-Brazilian-variants-harboring-mutations-e484k-and-n501y-in-the-spike-protein/585.

[B51] Nchioua R. Schundner A. Klute S. Koepke L. Hirschenberger M. Noettger S.. (2023). Reduced replication but increased interferon resistance of SARS-CoV-2 Omicron BA.1. Life Sci. alliance 6, e202201745–e202201745. doi: 10.26508/lsa.202201745, PMID: 36977594 PMC10053418

[B52] Ong S. W. X. Chiew C. J. Ang L. W. Mak T.-M. Cui L. Toh M. P. H. S.. (2021). Clinical and virological features of SARS-CoV-2 variants of concern: a retrospective cohort study comparing B.1.1.7 (Alpha), B.1.315 (Beta) and B.1.617.2 (Delta). Clin. Infect. Diseases: Off. Publ. Infect. Dis. Soc. America 75 (1), e1128–e1136. doi: 10.1093/cid/ciab721, PMID: 34423834 PMC8522361

[B53] Peacock T. P. Brown J. C. Zhou J. Thakur N. Newman J. Kugathasan R.. (2022). The altered entry pathway and antigenic distance of the SARS-CoV-2 Omicron variant map to separate domains of spike protein. bioRxiv. 2021.12.31.474653. doi: 10.1101/2021.12.31.474653v2

[B54] Plante J. A. Liu Y. Liu J. Xia H. Johnson B. A. Lokugamage K. G.. (2020). Spike mutation D614G alters SARS-CoV-2 fitness. Nature 592. doi: 10.1038/s41586-020-2895-3, PMID: 33106671 PMC8158177

[B55] Plante J. A. Mitchell B. M. Plante K. S. Debbink K. Weaver S. C. Menachery V. D. (2021). The variant gambit: COVID-19’s next move. Cell Host Microbe 29 (4). doi: 10.1016/j.chom.2021.02.020, PMID: 33789086 PMC7919536

[B56] Sentis C. Billaud G. Bal A. Frobert E. Bouscambert M. Destras G.. (2022). SARS-coV-2 omicron variant, lineage BA.1, is associated with lower viral load in nasopharyngeal samples compared to delta variant. Viruses 14, 919. doi: 10.3390/v14050919, PMID: 35632661 PMC9144383

[B57] Shuai H. Chan J. F.-W. Hu B. Chai Y. Yuen T. T.-T. Yin F.. (2022). Attenuated replication and pathogenicity of SARS-CoV-2 B.1.1.529 Omicron. Nature 603, 693–699. doi: 10.1038/s41586-022-04442-5, PMID: 35062016

[B58] Son K. Liang Z. Lipton H. L. (2015). Double-stranded RNA is detected by immunofluorescence analysis in RNA and DNA virus infections, including those by negative-stranded RNA viruses. J. Virol. 89, 9383–9392. doi: 10.1128/jvi.01299-15, PMID: 26136565 PMC4542381

[B59] Stauft C. B. Sangare K. Wang T. T. (2023). Differences in new variant of concern replication at physiological temperatures *in vitro* . J. Infect. Dis. 227, 202–205. doi: 10.1093/infdis/jiac264, PMID: 35759271 PMC9384407

[B60] Targett-Adams P. Boulant S. McLauchlan J. (2007). Visualization of Double-Stranded RNA in cells supporting hepatitis C virus RNA replication. J. Virol. 82, 2182–2195. doi: 10.1128/jvi.01565-07, PMID: 18094154 PMC2258944

[B61] van Dorp L. Acman M. Richard D. Shaw L. P. Ford C. E. Ormond L.. (2020). Emergence of genomic diversity and recurrent mutations in SARS-CoV-2. Infection Genet. Evol. 83, 104351. doi: 10.1016/j.meegid.2020.104351, PMID: 32387564 PMC7199730

[B62] Viana R. Moyo S. Amoako D. G. Tegally H. Scheepers C. Althaus C. L.. (2022). Rapid epidemic expansion of the SARS-CoV-2 Omicron variant in southern Africa. Nature 603, 679–686. doi: 10.1038/s41586-022-04411-y, PMID: 35042229 PMC8942855

[B63] Voloch C. M. da Silva Francisco R. de Almeida L. G. P. Cardoso C. C. Brustolini O. J. Gerber A. L.. (2021). Genomic characterization of a novel SARS-coV-2 lineage from rio de janeiro, Brazil. J. Virol. 95(10). doi: 10.1128/jvi.00119-21, PMID: 33649194 PMC8139668

[B64] Vu M. N. Lokugamage K. G. Plante J. A. Scharton D. Bailey A. O. Sotcheff S.. (2022). QTQTN motif upstream of the furin-cleavage site plays a key role in SARS-CoV-2 infection and pathogenesis. Proc. Natl. Acad. Sci. 119 (32). doi: 10.1073/pnas.2205690119, PMID: 35881779 PMC9371735

[B65] Weber F. Wagner V. Rasmussen S. B. Hartmann R. Paludan S. R. (2006). Double-Stranded RNA is produced by Positive-Strand RNA viruses and DNA viruses but not in detectab le amounts by Negative-Strand RNA viruses. J. Virol. 80, 5059–5064. doi: 10.1128/jvi.80.10.5059-5064.2006, PMID: 16641297 PMC1472073

[B66] Welsch S. Miller S. Romero-Brey I. Merz A. Bleck C. K. Walther P.. (2009). Composition and Three-Dimensional architecture of the dengue virus replication and assembly sites. Cell Host Microbe 5, 365–375. doi: 10.1016/j.chom.2009.03.007, PMID: 19380115 PMC7103389

[B67] Willett B. J. Grove J. MacLean O. A. Wilkie C. De Lorenzo G. Furnon W.. (2022). SARS-CoV-2 Omicron is an immune escape variant with an altered cell entry pathway. Nat. Microbiol. 7, 1161–1179. doi: 10.1038/s41564-022-01143-7, PMID: 35798890 PMC9352574

[B68] World Health Organization (2023). Updated working definitions and primary actions for SARSCoV2 variants. Available online at: https://www.who.int/publications/m/item/updated-working-definitions-and-primary-actions-for–sars-cov-2-variants (Accessed April 20, 2021).

[B69] World Health Organization (2025). COVID-19 epidemiological update. Available online at: https://www.who.int/publications/m/item/covid-19-epidemiological-update-edition-177 (Accessed May 09, 2025).

[B70] Zeng C. Evans J. P. Qu P. Faraone J. Zheng Y.-M. Carlin C.. (2021). Neutralization and stability of SARS-coV-2 omicron variant. bioRxiv. 2021.12.16.472934. doi: 10.1101/2021.12.16.472934v1

[B71] Zheng W. Cui L. Li M. Li Y. Fan W. Yang L.. (2021). Nucleoprotein phosphorylation site (Y385) mutation confers temperature sensitivity to influenza A virus due to impaired nucleoprotein oligomerization at a lower temperature. Science China. Life Sci. 64, 633–643. doi: 10.1007/s11427-020-1727-y, PMID: 32803713

[B72] Zimmermann L. Zhao X. Makroczyova J. Wachsmuth-Melm M. Prasad V. Hensel Z.. (2023). SARS-CoV-2 nsp3 and nsp4 are minimal constituents of a pore spanning replication organelle. Nat. Commun. 14 (1). doi: 10.1038/s41467-023-43666-5, PMID: 38036567 PMC10689437

