# Supplementary material for: Replication differences of SARS-CoV-2 lineages may arise from unique RNA replication characteristics and nucleocapsid protein expression
Source: Front Cell Infect Microbiol. 2025 Jul 11;15:1582137. doi: 10.3389/fcimb.2025.1582137 (PMC12289694; doi:10.3389/fcimb.2025.1582137)
Supplement: Supplementary file 1 [file SupplementaryFile1.docx]

**Supplementary Figure 1:** **Generation of viral stocks through passages in cell culture selected viruses with few mutations.** Mutations detected by NGS sequencing of the five variants of the study through cell passages are represented for SARS-CoV-2 genes Orf 1a gene, Orf 1b, the regions from spike gene: N terminal domain (NTD), receptor binding domain (RBD), furin cleavage site (FCS) and S2; nucleocapsid gene (N), structural genes - envelope (E) and membrane (M)- and ORFs (3a, 6, 7a, 7b, 8 and 9b). The substitutions in blue represent those detected only one time. Mutations in red represent those that were detected and fixed in the viral population. In parentheses, it indicated the passage number where the mutation was detected.


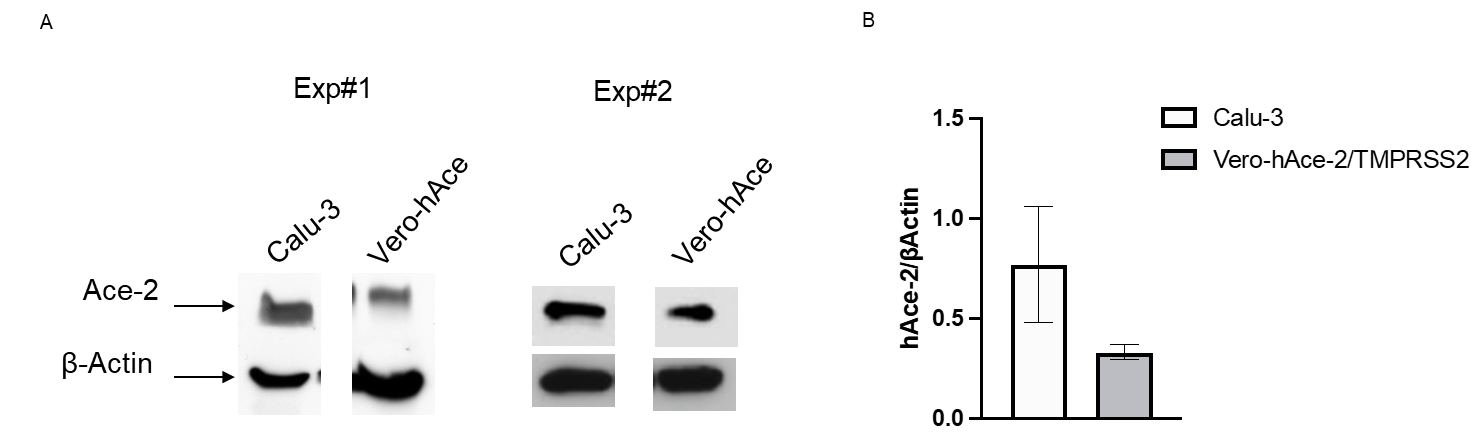


**Supplementary Figure 2:** **Expression levels of Ace-2 protein are similar in Calu-3 and Vero-hAce2/h-TMPRST2.** Cells (1x10^6^) were lysed and submitted to SDS-PAGE/WB with antibodies against human Ace-2 and β-actin. Western blotting images from two different experiments were quantified by ImageJ. Data represent the mean and standard deviation of the hAce-2/β-actin relationship from arbitrary values.

**
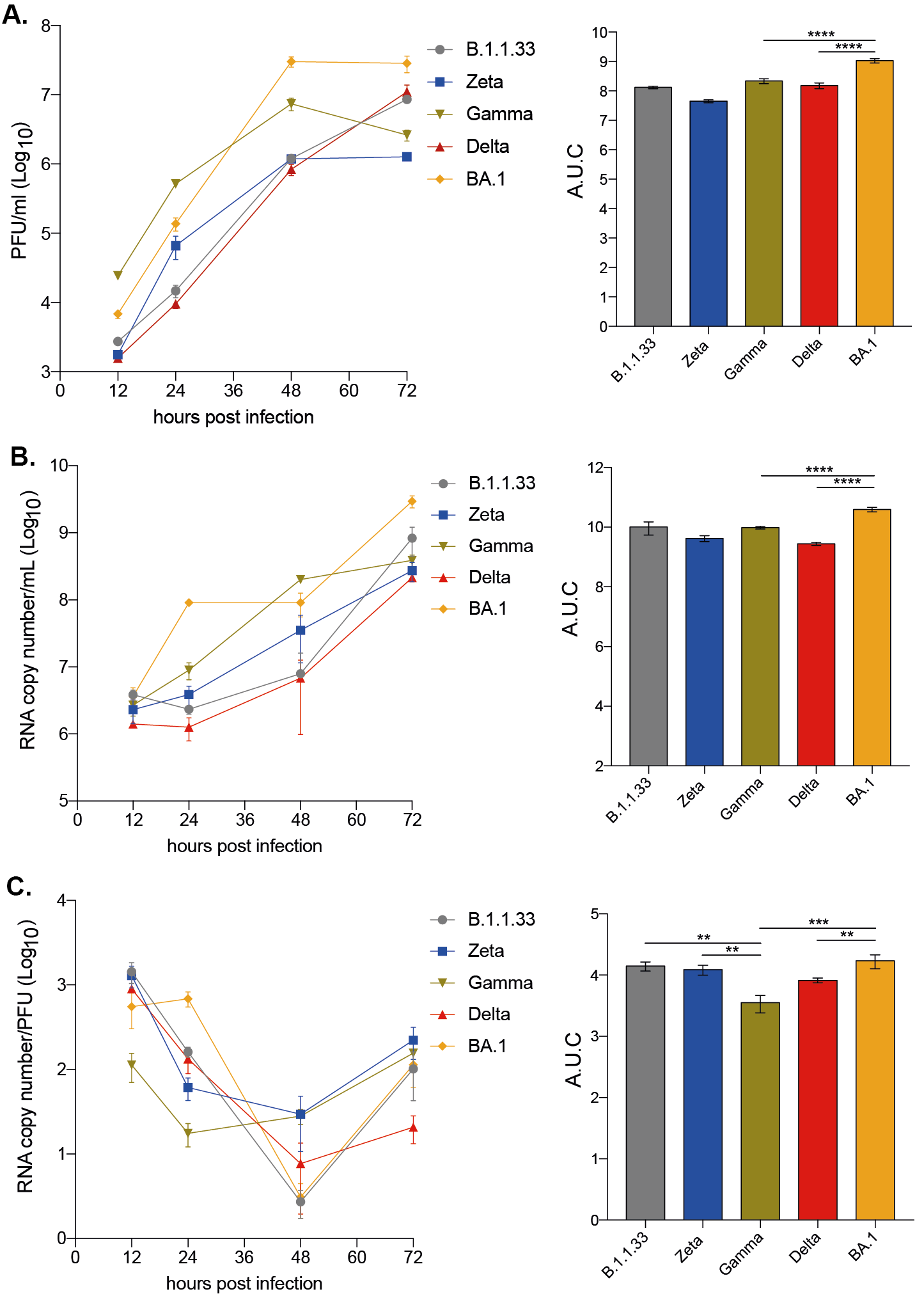
**

**Supplementary figure 3:** **BA.1 variant sustains its higher infection capacity in Vero E6 cells.** Cells were infected with viral variants at MOI 0.1 and incubated at 37°C with 5% CO_2_ for 12, 24, 48 or 72 hours to evaluate viral replication kinetics. **A-** Quantification of infectious viral progeny at the indicated time points by plaque assay. **B-** Quantification of viral RNA copy number at the indicated time points by RT-qPCR **C-** Particle to plaque-forming unit (P: PFU) was obtained as a measurement of viral infectivity. Vitral titres from (**A**), RNA copy numbers from (**B**), and P: PFU from (**C**) are also depicted as area under the curve (AUC).  Data are shown as mean±s.d. *n*=3. Statistical analysis of AUC was performed by one-way ANOVA followed by Dunnette´s multiple comparisons test; *p*<0.05 was considered to be statistically significant. **p<0.01; ***p<0;001; ****p<0.0001.


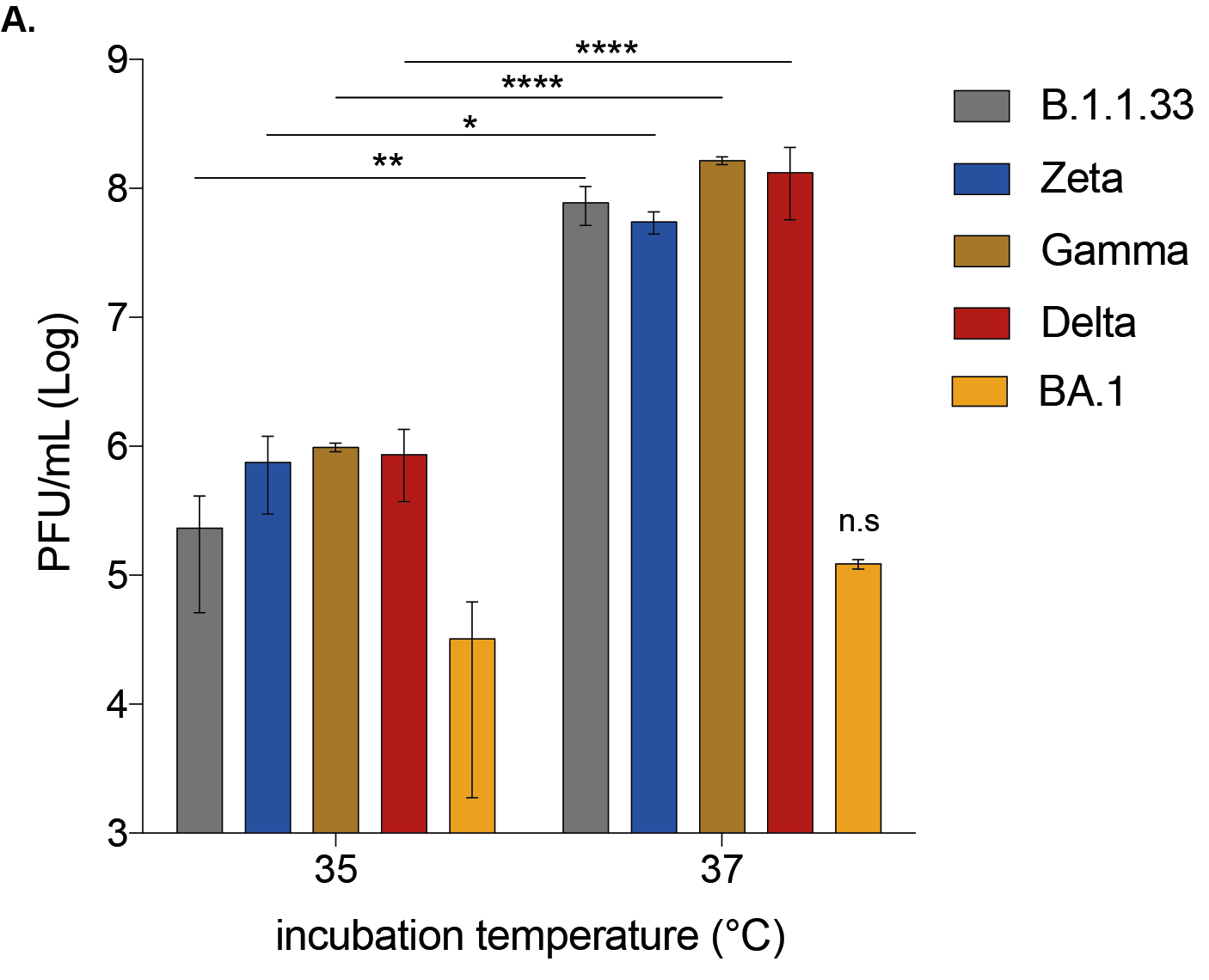


**Supplementary figure 4:** **Omicron BA.1 exhibits greater tolerance to lower infection temperatures in Calu-3 cells compared to other SARS-CoV-2 variants.** Calu-3 cells were infected with SARS-CoV-2 variants and incubated at 37°C for 1 hr for viral adsorption followed by 24 hours of incubation at 37°C or 35°C. The culture supernatant was harvested for viral titration by plaque assay. Data are shown as mean±s.d. *n*=3. Statistical analysis was performed by two-way ANOVA followed by Bonferroni's multiple comparisons test; *p*<0.05 was considered to be statistically significant. *p<0.05; **p<0.01; ****p<0.0001.


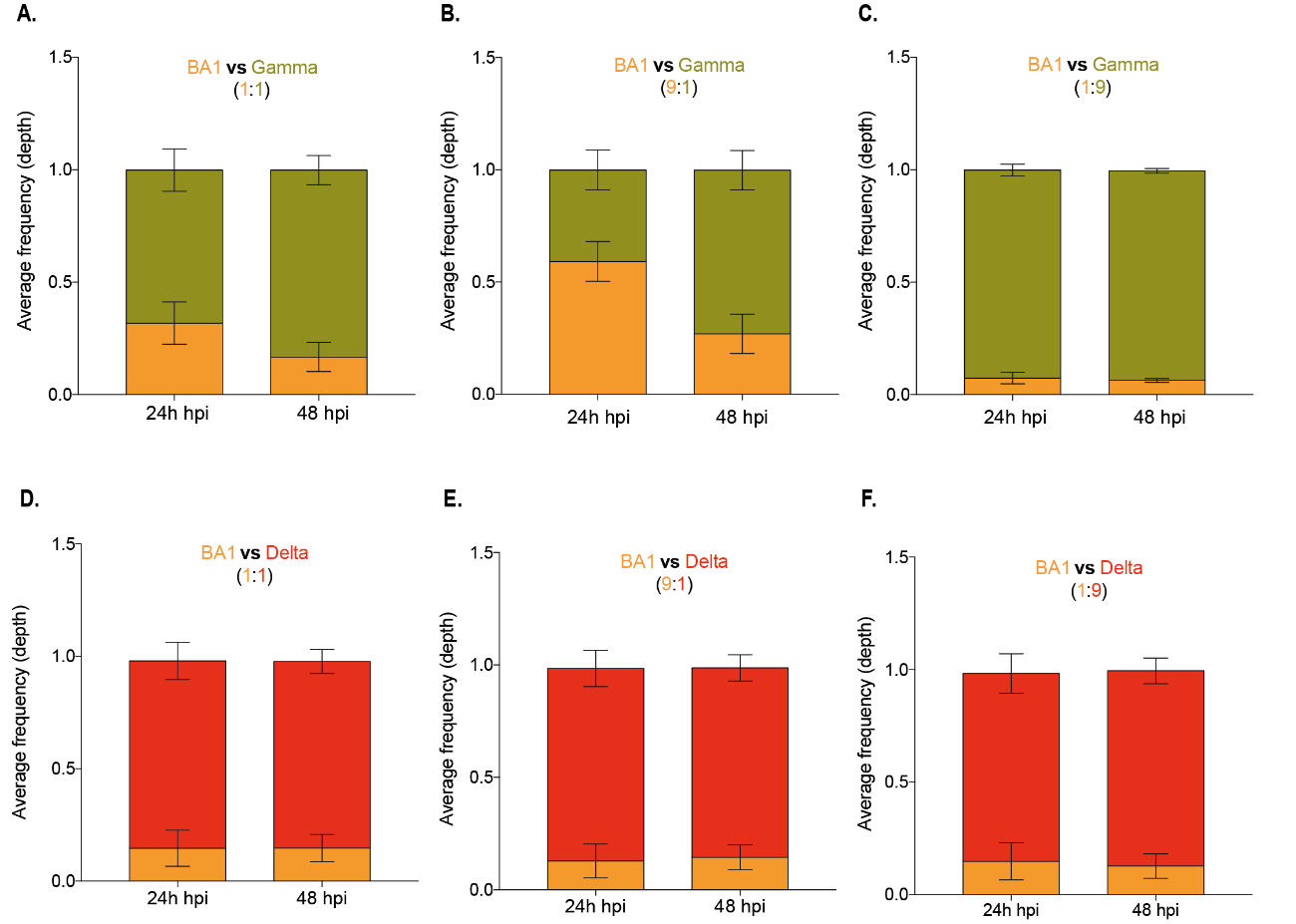


**Supplementary figure 5:** **Omicron BA.1 variant showed replication advantage in Vero/hACE-2/hTMPRSS2 cells according to time and viral proportion of infection.** Vero/hACE-2/hTMPRSS2 cells were co-infected with three different proportions of SARS-CoV-2 variants, resulting in a final MOI of 0.1 PFU. The infection was performed with variants Omicron BA.1 and Gamma at 1:1 **(A)**, 1:9 **(B)**, and 9:1 **(C)** proportions and with variants Omicron BA.1 and Delta at 1:1 **(D)**, 1:9 **(E)**, and 9:1 **(F)** proportions. Total RNA was extracted from culture supernatant 24- or 48-hours post-infection and subjected to NGS sequencing. The depth of each genomic region from viral lineages was plotted as the average frequency of detected sequences. Bar colors represent viral lineages BA.1 (yellow), Gamma (green), and Delta (red).


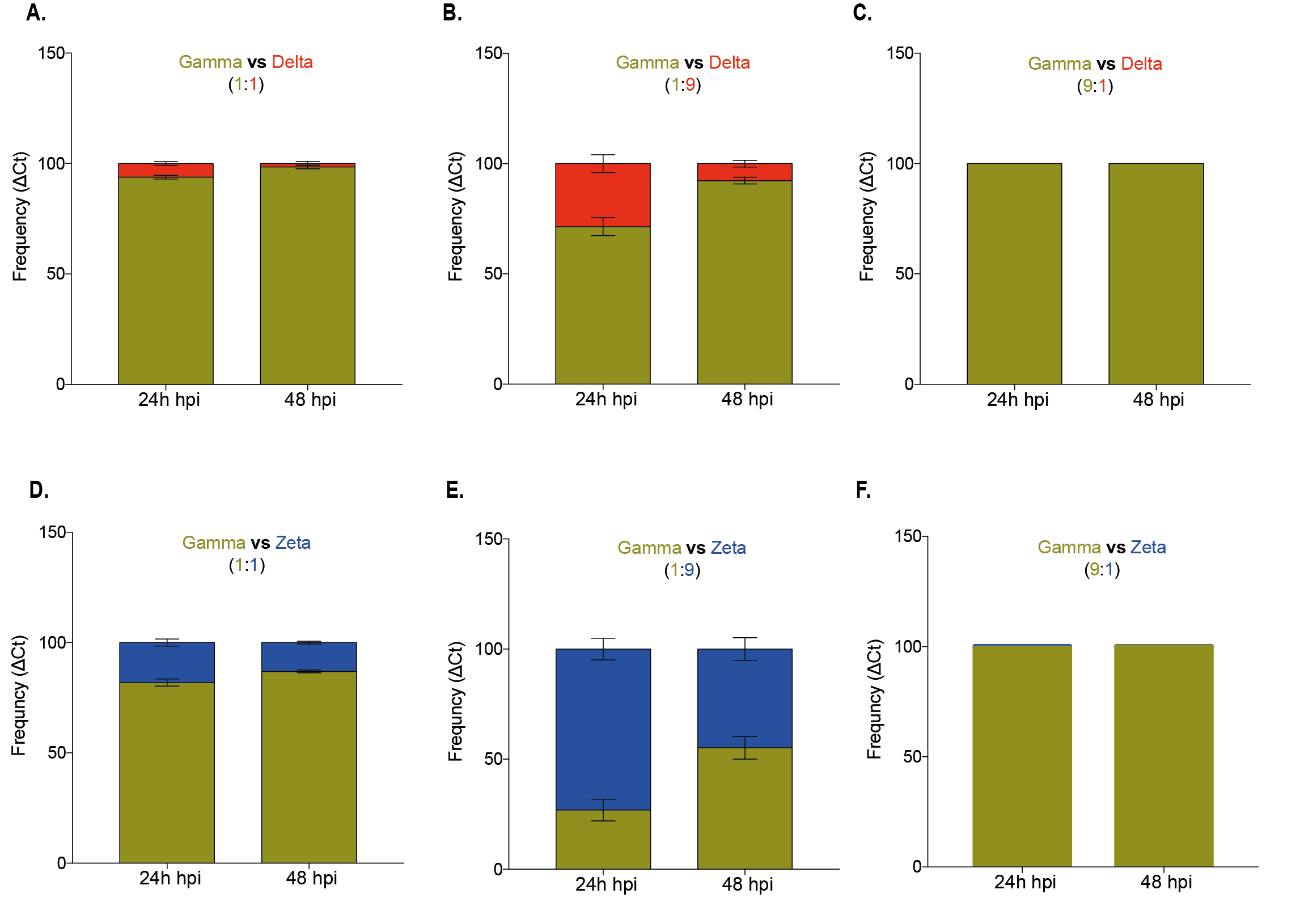


**Supplementary figure 6:** **SARS-CoV-2 Gamma variant is predominant in competition assay in Calu-3 cells.** Calu-3 cells were co-infected with three different proportions of SARS-CoV-2 variants resulting in a final MOI of 0.1 PFU. The infection was performed with variants Gamma and Delta at 1:1 **(A)**, 1:9 **(B)** and 9:1 **(C)** proportions; and variants Gamma and Zeta at 1:1 **(D)**, 1:9 **(E)** and 9:1 **(F)** proportions. Total RNA was extracted from culture supernatant after 24 or 48 hours post infection and subjected to RT-qPCR for variant detection through specific primers. The ΔCt value was used to calculate the percentage of each lineage. Bar colors represent viral lineages Gamma (green), Delta (red) and Zeta (blue).


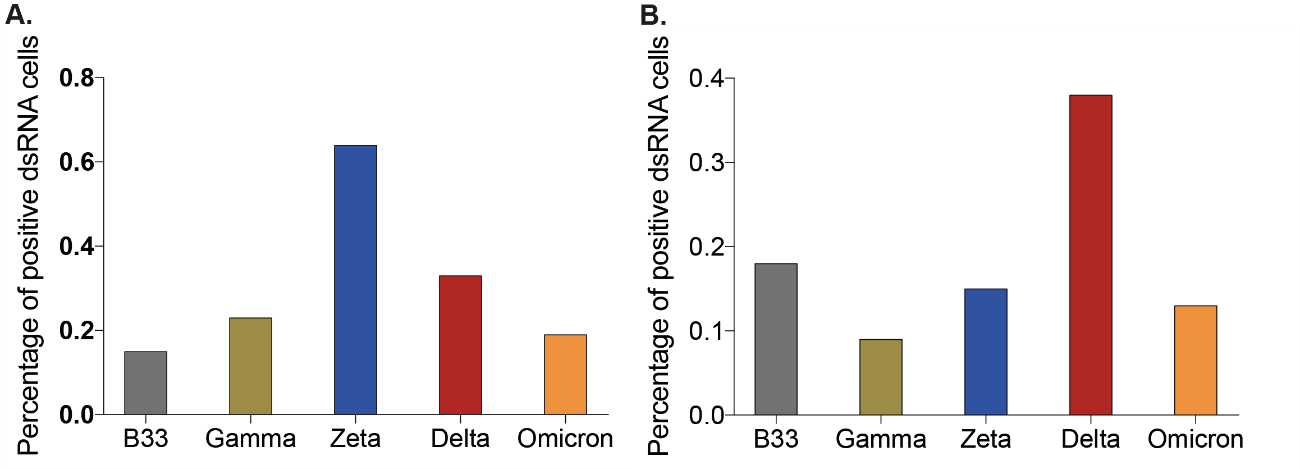


**Supplementary Figure 7:** **Percentage of positive dsRNA lung cells infected by SARS-CoV 2 VOC's.** **(A-B)** Percentage of Calu-3 with dsRNA positive puncta according to different tested VOCs at 8 and 24 hours post-infection, respectively. Representative data of 25 different analyzed fields corresponding to n ≥ 700 cells for each time point and VOC's.


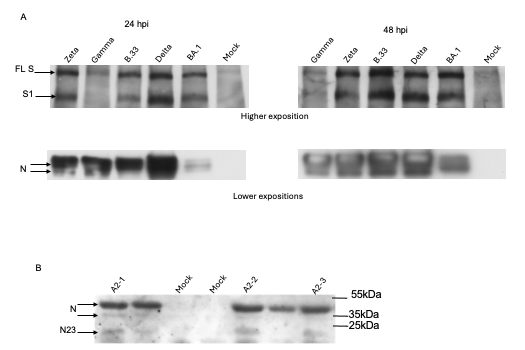


**Supplementary figure 8:** **Production of viral proteins S and N. A-** Higher exposition of S proteins from figure 8A. **B-** Calu-3 cells infected with a SARS-CoV-2 introduced in march 2020 in Brazil (lineage A2) was lysed after 24 hours post infection and submitted to SDS-PAGE/WB with antibodies against viral N protein. Arrows indicated the full-length protein (N) and its isoforms (N23).

**Supplementary Figure 9:** **N protein alignment of SARS-CoV-2 variants reveals specific amino acids for Delta at the linker region.** Sequences of nucleocapsid gene from viral stocks, original SARS-CoV-2 viruses (Wuhan), and a virus isolated at the beginning of the pandemic in Rio de Janeiro (A2 - GISAID # 528539) were aligned using BioEdit. Translation of sequences illustrates the amino acids of each N region, highlighted for Wuhan sequence: N- terminal domain (NTD) in red, RNA binding domain in yellow, linker region in green, protein oligomerization region in blue and C- terminal domain (CTD) in purple. Asterisk in red and gray amino acids indicates where protein cleavage occurs based on the study of Lutomski and Meyer, respectively.
